# Uncovering biomarkers and molecular pathways linking NAFLD and AIS: Insights from bioinformatic analysis and experiment

**DOI:** 10.1371/journal.pone.0333719

**Published:** 2025-09-29

**Authors:** Junjie Su, Fenglei Sun

**Affiliations:** 1 The First Clinical Medical College, Shandong University of Traditional Chinese Medicine, Jinan, Shandong Province, China; 2 Affiliated Hospital of Shandong University of Traditional Chinese Medicine, Jinan, Shandong Province, China; The University of Sheffield, UNITED KINGDOM OF GREAT BRITAIN AND NORTHERN IRELAND

## Abstract

**Background:**

Non-alcoholic fatty liver disease (NAFLD) and acute ischemic stroke (AIS) are both closely related to chronic inflammation and metabolic disorders, but the molecular mechanisms between them are not yet clear. This study aims to explore the common molecular characteristics between NAFLD and AIS using bioinformatics methods.

**Methods:**

We integrated transcriptomic data from GEO (GSE89632 for NAFLD and GSE16561 for AIS) to identify shared molecular signatures. Differentially expressed genes (DEGs) were screened using the limma package, and co-expression modules were identified via WGCNA. Functional enrichment and immune infiltration analyses were conducted using standard bioinformatics tools. Key genes were selected through LASSO and random forest algorithms, and candidate drugs were predicted using the CMap database. Experimental validation included qPCR, immunohistochemistry, and drug intervention in HepG2 and HMC3 cell models, as well as a high-fat diet-induced NAFLD mouse model.

**Results:**

Through differential expression analysis, 65 common DEGs were identified in both NAFLD and AIS. Functional enrichment analysis showed that these genes mainly involve signal pathways related to immune regulation and inflammatory responses. Immune infiltration analysis showed a significant increase in monocytes, B cells, and plasma cells in NAFLD patients. Ultimately, four potential biomarkers were screened out using LASSO regression and random forest algorithms: *CEBPD*, *SOCS2*, *THBS1*, and *IFIT2*. Using cMAP, 10 candidate therapeutic drugs, including lamotrigine, were identified. Expression of *CEBPD* and *SOCS2* was consistently upregulated in NAFLD clinical datasets, FFA-treated HepG2 cells, and liver tissues of HFD-induced NAFLD mice, but not in AIS datasets or OGD-treated microglial cells. Furthermore, lamotrigine, cinnarizine, and lenvatinib significantly suppressed FFA-induced *CEBPD* and *SOCS2* expression in HepG2 cells (*p* < 0.05), supporting their potential regulatory effects.

**Conclusions:**

This study identified common DEGs between NAFLD and AIS and experimentally validated *CEBPD* as a potential marker, revealing possible common molecular mechanisms between the two diseases, providing new directions for future diagnosis and treatment.

## 1 Introduction

Non-alcoholic fatty liver disease (NAFLD) is a complex metabolic disorder with a global prevalence of 38% among adults and approximately 10% among children and adolescents. This makes it a significant public health issue that impacts quality of life and economic stability [1]. Current NAFLD treatments focus on regulating glucose and lipid metabolism, improving lifestyle, and adjusting diet, yet outcomes remain unsatisfactory [2,3]. About 10% of NAFLD cases progress to cirrhosis and liver cancer. NAFLD is linked to various diseases, including metabolic syndrome, diabetes, hyperlipidemia, and hypertension, all of which are risk factors for stroke [4].

In clinical practice, the prognosis of NAFLD varies significantly among patients. Some remain at high risk for cerebrovascular disease despite lipid-lowering and liver-protective treatments. A study involving 79,905 participants found that the risk of stroke during follow-up was 16% higher in the NAFLD group compared to the non-NAFLD group (95% CI: 1.07 to 1.26), and the severity of NAFLD correlates with the risk of ischemic stroke [5].

Ischemic stroke (IS) is the most common severe manifestation of cerebrovascular disease, with increasing incidence among younger populations [6]. It accounts for 87% of all stroke subtypes in the United States and 63% globally, making it a leading cause of death and severe disability. The burden of stroke is rising, particularly in low-income countries [7–9].

Recent studies indicate that, while NAFLD is primarily a metabolic disorder, it also involves multiple immune-mediated inflammatory processes. Certain liver diseases are closely related to neurological disorders through the “hepato-cerebral axis,” with mechanisms including blood-brain barrier (BBB) permeability, vagus nerve activity, epigenetic regulation, toxic metabolites, β-amyloid (Aβ) metabolism, and immune responses [10]. Although current research has highlighted the link between NAFLD and acute ischemic stroke (AIS) through inflammation, further investigation is necessary to explore whether immune-mediated inflammatory pathways in NAFLD patients increase their susceptibility to acute cerebral infarction and whether genetic factors play a role [11].

This study aims to identify key genes associated with the pathogenesis of both NAFLD and AIS, investigate potential molecular mechanisms, and explore small molecule compounds that could be potential therapeutic agents. Using R language, we will determine differentially expressed genes (DEGs) and their functional roles in both conditions. Through immune infiltration analysis and machine learning techniques, we plan to identify important genes, develop a diagnostic model, and predict potential small molecule compounds for the diagnosis and prevention of these diseases, ultimately providing valuable insights into their shared pathogenesis.

## 2 Methods

### 2.1 Data source and preprocessing

In the GEO platform, we selected the NAFLD dataset GSE89632 [12] based on platform GPL14951 and the AIS dataset GSE16561 [13] based on platform GPL6883. GSE89632 includes 63 participants (20 with simple steatosis, 19 with nonalcoholic steatohepatitis, and 24 healthy controls), focusing on hepatic gene expression and fatty acid composition using the Illumina Microarray platform. GSE16561 profiles peripheral blood gene expression in 39 acute ischemic stroke patients and 24 healthy controls. Both datasets have been widely used in published studies and offer well-defined cohorts, making them suitable for investigating the common molecular pathways between NAFLD and AIS. The collected microarray data were preprocessed in RStudio, where gene expression matrices were read, normalized, and ultimately resulted in gene expression profile files. The GSE89632 and GSE16561 datasets were organised using the tidyverse and limma packages in R Studio. Based on their corresponding platform files, the ID and SYMBOL columns of the platform files were extracted, missing data were removed, and issues with a single probe corresponding to multiple genes were addressed. After removing redundant columns, the gene expression profiles were obtained. Before conducting our analysis, we performed adjustment for potential confounding factors, including age and gender, by including them as covariates in our statistical models. Importantly, because GSE89632 (liver tissue) and GSE16561 (blood samples) are derived from distinct tissues and platforms, we analyzed them independently and identified overlapping DEGs across datasets, rather than merging raw expression matrices. Therefore, cross-dataset batch correction was not applied. In addition, the NAFLD datasets GSE58979, GSE63067 and the AIS dataset GSE22255 [14] was chosen as an external validation dataset. Details of the five datasets are provided in Table 1.

**Table 1:** Details regarding the 5 datasets.

Datasets	Platforms	Type of Samples	Control sample size	NAFLD or AIS sample size	Applications
**GSE89632**	GPL14951	NAFLD	24	39	Discovery of DEGs
**GSE16561**	GPL6883	AIS	24	39	Discovery of DEGs
**GSE58979**	GPL15207	NAFLD	28	25	Validation of hub genes
**GSE63067**	GPL570	NAFLD	7	11	Validation of hub genes
**GSE22255**	GPL570	AIS	20	20	Validation of hub genes

The type of platforms, numbers of samples, samples application types.

### 2.2 Differential expression gene analysis

Using the limma package in RStudio, differential analysis was performed with the lmFit and eBayes functions. The screening criteria were set at |logFC| > 0.585 and an adjusted P-value < 0.05. Significant DEGs in the disease group and healthy group from GSE89632 and GSE16561 were identified. Heatmaps and volcano plots were generated for the DEGs in both NAFLD and AIS. Finally, a Venn diagram was created to display the common DEGs between NAFLD and AIS.

### 2.3 WGCNA analysis

Using the Weighted gene co-expression network analysis(WGCNA) software package, the gene expression profile data of both datasets were processed. We read the sample information for each experimental group and control group, conducted data preprocessing, and calculated the correlation of gene expression levels using Pearson’s correlation coefficient. The top 5000 genes based on the median absolute deviation (MAD) were selected. The goodSamplesGenes function was employed to exclude low-quality samples or genes, and after identifying outliers, we reclustered the samples and used WGCNA to identify co-expression modules. Genes with similar expression profiles were clustered into gene modules through TOM similarity (adjacency). We then compiled clinical information to derive the correlation between gene modules and clinical data.

### 2.4 Functional enrichment analysis

Using an adjusted P-value filter of <0.05 as the selection criterion, we performed functional enrichment analyses on the DEGs and common genes (CGs) for both NAFLD and AIS. Gene Set Enrichment Analysis (GSEA) was performed to identify significantly enriched pathways associated with the candidate genes. Gene expression profiles were ranked based on differential expression between groups, and the enrichment of predefined gene sets from the KEGG databases was evaluated. Pathways with a false discovery rate (FDR) < 0.25 were considered significantly enriched [15].

### 2.5 Immune cell infiltration analysis

The CIBERSORT algorithm was utilized to analyze the differences in the proportions of 22 immune cell types in the NAFLD expression matrix. A P-value < 0.05 was set as the filtering criterion for the results obtained, allowing us to identify differences in immune cell proportions between the NAFLD group and the healthy control group. Visualization of the analysis was performed using ggplot2.

### 2.6 Machine learning

To identify key genes associated with the diseases, we performed LASSO (Least Absolute Shrinkage and Selection Operator) regression analysis, which is a penalized regression method used to enhance the prediction accuracy and interpretability of the model. The LASSO model was applied with 10-fold cross-validation to select the optimal penalty parameter (λ), minimizing the model’s complexity while avoiding overfitting. The genes that remained after the LASSO shrinkage were considered the most relevant to NAFLD and AIS.

Following this, we utilized the “RandomForest” package to analyze the selected genes, setting the number of trees to 100 and using default parameters for splitting. Gene importance was evaluated based on the mean decrease in Gini index. The combination of LASSO and random forest enhanced both feature selection and model robustness.

### 2.7 Prediction of potential small molecule compounds

Using the CMap platform, we submitted the common DEGs for searching and downloaded the results. We filtered for perturbation types labeled as trt-cp and removed any results with missing values. The remaining results were ranked based on normalized drug scores and significance, selecting potential drugs with high composite rankings.

### 2.8 Cell culture and drug intervention

HepG2 cells were cultured in Dulbecco’s Modified Eagle Medium (DMEM) supplemented with 10% fetal bovine serum (FBS) and 1% penicillin-streptomycin, maintained in a 37°C humidified incubator with 5% CO₂. For the free fatty acid (FFA) intervention, cells were seeded in 6-well plates and allowed to reach approximately 80% confluence. To induce lipid accumulation, cells were treated with a mixture of palmitic acid (PA) and oleic acid (OA) at a final concentration of 1 mM, prepared in serum-free medium. After a 24-hour incubation, cells were harvested for subsequent assays. Control cells were treated with serum-free medium without FFAs.

The human microglial cell line HMC3 was routinely cultured in DMEM/F12 supplemented with 10% FBS and 1% penicillin-streptomycin, and maintained at 37°C in a humidified 5% CO₂ incubator. Cells were passaged at 80–90% confluence for experimental use. For oxygen-glucose deprivation (OGD) experiments, cells were seeded at appropriate densities and allowed to adhere overnight. Prior to OGD induction, the culture medium was removed and cells were carefully washed twice with PBS to eliminate residual glucose and serum components. The glucose-free treatment was initiated by replacing the medium with pre-equilibrated glucose-free DMEM, after which the culture plates were immediately transferred to a hypoxia chamber flushed with a gas mixture of 1% O₂, 5% CO₂, and 94% N₂. The chamber was sealed and placed in a 37°C incubator for the designated treatment period, typically ranging from 8 hours.

The study included five groups: a control group cultured under normal conditions, a model group subjected to specific disease/injury induction, and three drug-treated groups receiving 50 μM lamotrigine (84057-84-1, MCE), 10 μM cinnarizine (298-57-7,MCE), or 100 nM lenvatinib (417716-92-8, MCE). All drugs were dissolved in DMSO with a final concentration not exceeding 0.1%, and vehicle control (0.1% DMSO) was included in parallel. After cell attachment, the culture medium was replaced with fresh medium containing the corresponding drugs or vehicle, followed by 24-hour incubation.

### 2.9 Transcription factor prediction

The potential transcription factors regulating the core genes were predicted using the hTFtarget database (S1A, B Table).

### 2.10 Single-cell analysis

Liver scRNA-seq data were obtained from the GEO dataset GSE115469 (platform: GPL16791). Quality control was performed to remove low-quality cells, excluding those with fewer than 200 detected genes, more than 6,000 genes, or mitochondrial gene content exceeding 10%. Genes expressed in fewer than 3 cells were also discarded. Counts were normalized and log-transformed, highly variable features were identified, and data were scaled while regressing out sequencing depth and mitochondrial percentage. PCA was performed for initial dimensionality reduction, and t-SNE was run on the top principal components (dims = 1:10) with a perplexity of 30 and 1,000 iterations (random seed = 42). Plots were generated with Seurat (v4) and colored by gene expression levels, including visualization of key genes such as *CEBPD* and *SOCS2*.

### 2.11 qPCR Validation

Total RNA was extracted using a standard TRIzol method [16]. The purity and concentration of the RNA were assessed using a NanoDrop spectrophotometer. cDNA was synthesized from 1 µg of RNA using a reverse transcription kit according to the manufacturer’s protocol. qPCR was performed using SYBR Green Master Mix on an ABI 7500 real-time PCR system. The target genes validated in this study included *CEBPD*, *SOCS2*, *THBS1*, and *IFIT2*, with *β-Actin* used as an internal control. The primer sequences are shown in Table 2.

**Table 2:** The primer sequences.

	Forward	Reverse
***CEBPD***	TCGACTTCAGCGCCTACATC	CTTTGCGCTCCTATGTCCCA
***SOCS2***	GAGCCGGAGAGTCTGGTTTC	ATCCTGGAGGACGGATGACA
***IFIT2***	CACCTCTACTCTGCCCTCCT	ACCAGGTTCCCACGTTTTGT
***THBS1***	CAGGAGCAACCTCTACTCCG	CAGCAGGGATCCTGTGTGTA

### 2.12 Western blot

Total protein was extracted from cells/tissues using RIPA buffer with protease inhibitors, quantified by BCA assay, separated by SDS–PAGE, and transferred onto PVDF membranes. Membranes were blocked with 5% non-fat milk and incubated overnight at 4 °C with primary antibodies against *CEBPD* and *SOCS2*, followed by HRP-conjugated secondary antibodies. Signals were visualized using ECL and captured on a chemiluminescence system. Band intensities were quantified with ImageJ and normalized to *GAPDH*.

### 2.13 Animal preparation

Male C57BL/6 mice (8 weeks old) were randomly divided into normal control (NC) group and NAFLD model group (n = 6/group). The NC group received standard chow diet, while the NAFLD group was fed a high-fat diet (HFD, 60% kcal fat, D12492; Research Diets) for 16 weeks to induce steatohepatitis. All animals were housed under controlled conditions (22 ± 2°C, 12-h light/dark cycle) with free access to food/water. The experimental protocol was approved by the Animal Ethics Committee of Shandong University of Traditional Chinese Medicine.

### 2.14 Immunohistochemical (IHC)

Paraffin-embedded liver sections (4μm) from NAFLD and control mice were deparaffinized and subjected to antigen retrieval in citrate buffer (pH6.0). After blocking endogenous peroxidase and non-specific binding, sections were incubated overnight at 4°C with anti-SOCS2 (1:200, DF8133, Affinity) or anti-CEBPD (1:150, AF9027, Affinity) antibodies, followed by HRP-conjugated secondary antibody and DAB development. Hematoxylin counterstaining was performed before mounting. Positive staining was analyzed using ImageJ software by measuring integrated density normalized to tissue area.

### 2.15 Statistical analysis

Statistical analysis was conducted using IBM SPSS Statistics version 26. Data from the two groups were compared, with results expressed as mean ± standard deviation (SD). Group differences were assessed using independent-samples t-tests, with a significance level set at *p* < 0.05.

## 3 Results

### 3.1 Identification of DEGs

**Fig 1:**
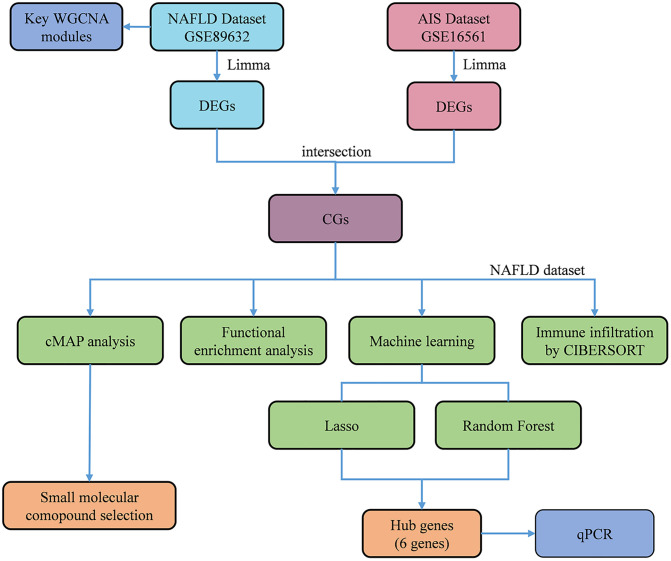
Flow chart of the analysis steps in this study.

**Fig 2:**
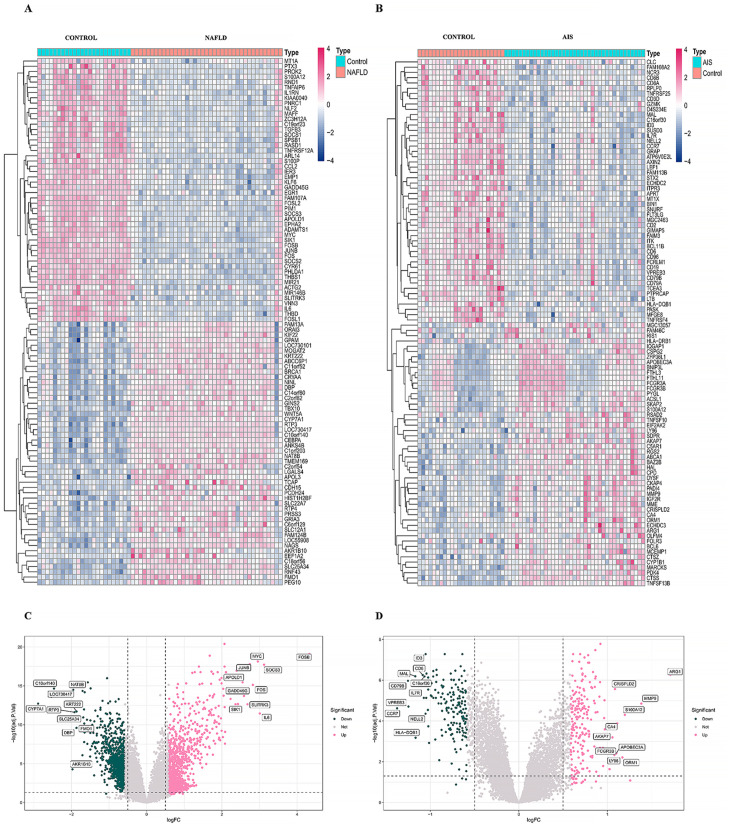
Differential expression analysis of the NAFLD and AIS datasets. (A) The top 50 differentially expressed genes (DEGs) are shown in the heatmap. (B) The top 50 DEGs are presented in the heatmap. (C) Volcano map of DEGs in NAFLD. (D) Volcano map of DEGs in AIS.

The analysis strategy of this study is illustrated in the flowchart (Fig 1). In the NAFLD dataset GSE89632, which includes 39 participants with NAFLD and 24 healthy controls, a total of 2,079 genes were identified as associated with NAFLD. The DEGs are shown in the heatmap and the volcano plot (Fig 2A, C). The AIS dataset GSE16561 contains data from 39 ischemic stroke patients and 24 healthy controls, with 309 genes identified as DEGs related to AIS. The top 50 DEGs are presented in the heatmap (Fig 2B). Upregulated DEGs include *ARG1*, *MMP9*, *S100A12*, *ORM1*, *APOBEC3A*, *CRISPLD2*, *CA4*, AKAP7, FCGR3B, and LY96. Conversely, the expression levels of the ten genes *hID3*, *CD6*, *MAL*, *C16orf30*, *IL7R*, *CD79B*, *VPREB3*, *CCR7*, *HLA − DQB1*, and *NELL2* were significantly downregulated in AIS patients compared to the healthy control group, as shown in the volcano plot (Fig 2D).

### 3.2 WGCNA analysis

**Fig 3:**
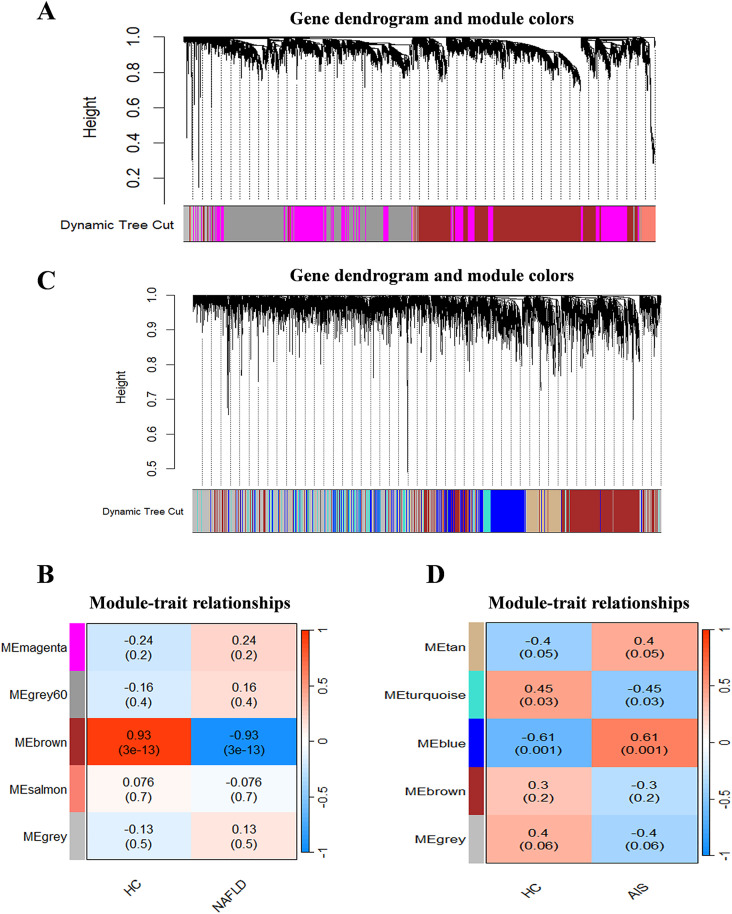
WGCNA. (A) Dendrogram of gene clustering in NAFLD, with different colors representing different modules. (B) Heatmap of the correlation between module characteristic genes and NAFLD; blue indicates negative correlation and red indicates positive correlation. (C) Dendrogram of gene clustering in AIS, with different colors representing different modules. (D) Heatmap of the correlation between module characteristic genes and AIS; blue indicates negative correlation and red indicates positive correlation.

Using the expression profile data from GSE89632, we further constructed a WGCNA network to explore the co-expression networks associated with NAFLD [17]. The TOM matrix was utilized to identify gene modules, resulting in the discovery of five gene modules, as depicted in the dendrogram (Fig 3A). These modules are designated as MEmagenta, MEgrey60, MEbrown, MEsalmon, and MEgrey, with the MEbrown module exhibiting the strongest inverse relationship with the disease (cor = 0.93, p = (3e - 13)) (Fig 3B). Similarly, applying the same methodology to the GSE16561 dataset revealed five gene modules: MEtan, MEturquoise, MEblue, MEbrown, and MEgrey, with the MEblue module showing the strongest inverse relationship with the disease (cor = 0.61, *p* = (0.001)) (Fig 3C, D).

### 3.3 Functional enrichment analysis

**Fig 4:**
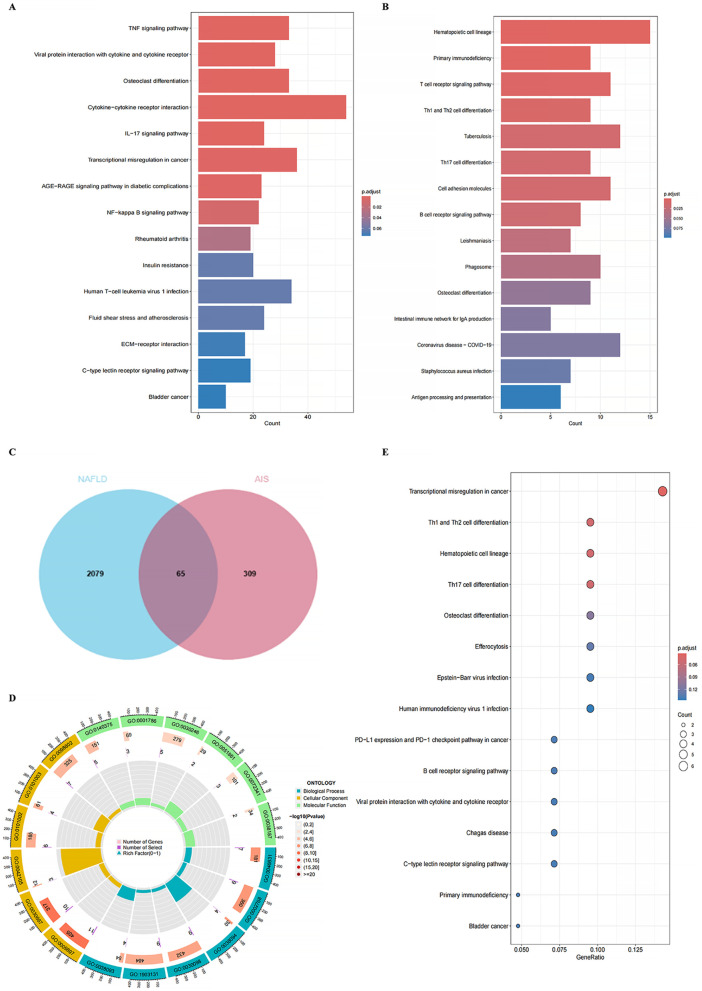
Functional enrichment analysis. (A) The results of KEGG enrichment analysis based on NAFLD. (B) The results of KEGG enrichment analysis based on AIS. (C) Venn diagram showing the intersection of CGs in NAFLD and AIS. (D) Circular plot of the results of GO enrichment analysis based on CGs. (E) The results of KEGG enrichment analysis based on CGs.

**Fig 5:**
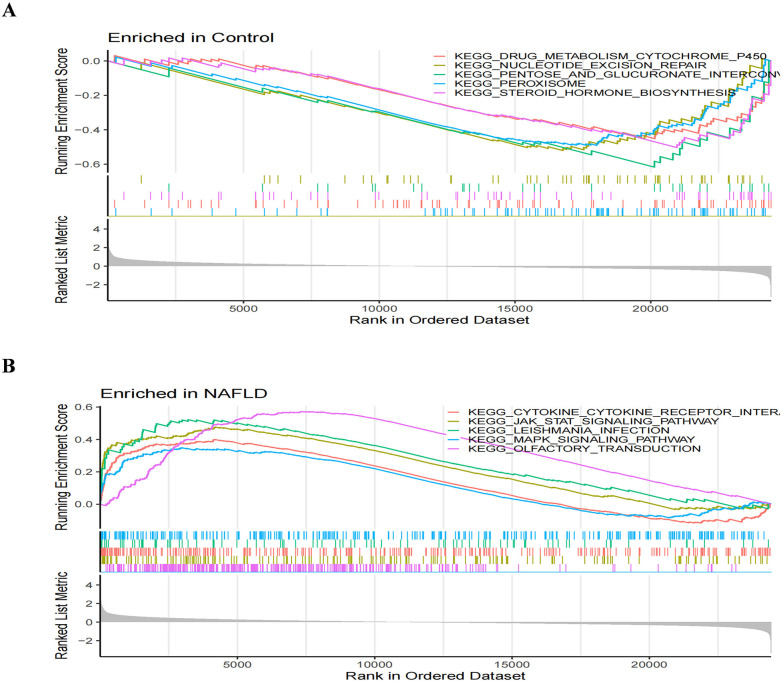
The results of GSEA.

The differential genes for NAFLD were analyzed in the KEGG database, revealing the top 15 pathways, as shown in (Fig 4A). Among these, the Cytokine−cytokine receptor interaction pathway exhibited the highest enrichment and the largest number of genes. For AIS, most genes were highly enriched in the Hematopoietic cell lineage pathway (Fig 4B). A Venn diagram generated using the web tool jvenn (inrae.fr) depicted a total of 65 common differential genes associated with both NAFLD and AIS (Fig 4C). We conducted GO and KEGG enrichment analyses on these 65 genes using the clusterProfiler and org.Hs.e.g.,db packages in R. The GO results yielded a total of 526 GO terms, including 441 biological processes (BP), 51 molecular functions (MF), and 34 cellular components (CC), with the top six most significantly enriched terms extracted for each category, as shown in the circle diagram (Fig 4D). KEGG analysis (Fig 4E) indicated that pathways such as Th1 and Th2 cell differentiation, Th17 cell differentiation, and Hematopoietic cell lineage significantly influence cell regulatory differentiation and involve various cytokines, most of which are related to inflammatory immune regulation [18].We confirmed the functional enrichment of genes in relevant biological pathways through gene set enrichment analysis (GSEA).NAFLD was enriched in the cytokine-cytokine receptor interaction, JAK-STAT signaling pathway, leishmania infection pathway, MAPK signaling pathway and olfactory transduction pathway.The KEGG pathways involved in the control group include the drug metabolism cytochrome p450, the nucleotide excision repair, pentose and glucuronate interconversions, peroxisomes, and steroid hormone biosynthesis(Fig 5A, B).

### 3.4 Immune infiltration analysis

**Fig 6:**
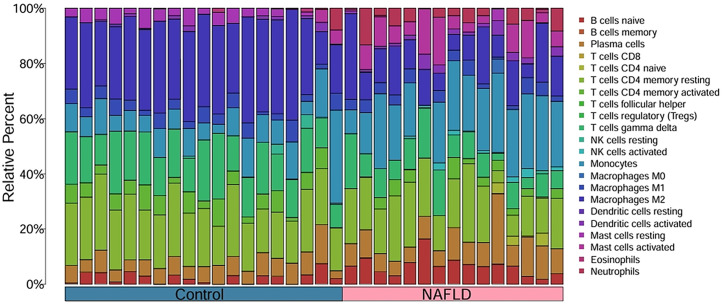
Analysis of immune cell infiltration in NAFLD.

Based on the functional and pathway analysis of the common differential genes in NAFLD and AIS, a close relationship with inflammatory and immune processes was identified [19]. To investigate the correlation of these differential genes with immunity, we employed the CIBERSORT algorithm to infer the characteristics of immune cell types. As illustrated in Fig 6, the proportions of 22 immune cell types in the HC group and NAFLD group from GSE89632 were displayed. Compared to the HC group, the NAFLD group showed an increased proportion of B cells naïve, Plasma cells, activated NK cells, Monocytes, activated Dendritic cells, activated Mast cells, and Neutrophils, while there was a decrease in the proportion of CD4 memory activated T cells, gamma delta T cells, M2 Macrophages, and resting Mast cells.

### 3.5 Machine learning and diagnostic model establishment

**Fig 7:**
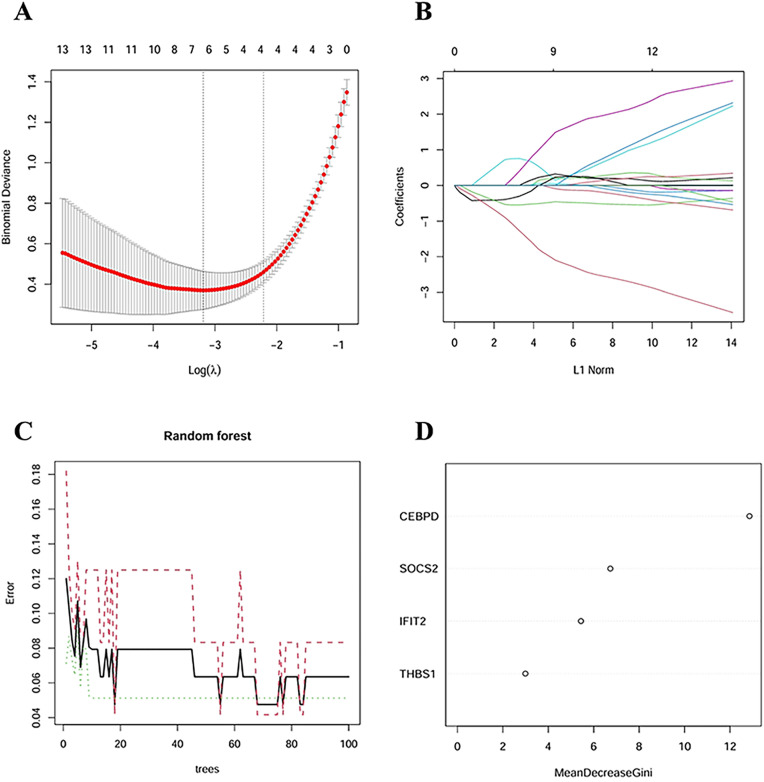
Screening facilitated the identification of diagnostic biomarkers using machine learning methods. (A,B) Lasso regression analysis. (C,D) The random forests result in a gene importance plot.

LASSO regression was employed to further screen the differential genes, beginning with the LASSO cross-validation curve (Fig 7A). We selected Log(λ) = 4 and compared it with the LASSO coefficient plot (Fig 7B). This process ultimately identified six potential genes that significantly affect the diagnosis of AIS in NAFLD patients: *CEBPD*, *SOCS2*, *THBS1*, *IFIT2*, *TNFSF10*, and *IL2RB*. Subsequently, we utilized random forests to rank the importance of these genes, resulting in a gene importance plot (Fig 7C, D) that led to the final selection of *CEBPD* and *SOCS2* as key genes. The earlier LASSO regression and random forest screenings facilitated the identification of diagnostic biomarkers.

### 3.6 Prediction of potential small molecule compounds

**Fig 8:**
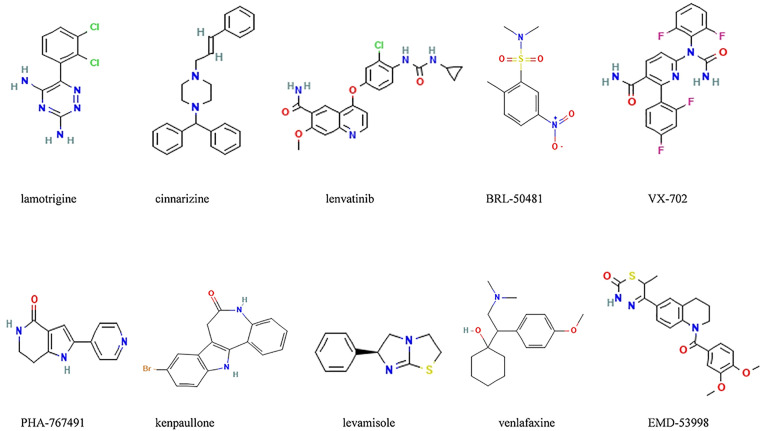
Screening of the potential small-molecular compounds for the treatment of NAFLD and AIS by cMAP analysis.

The common differential genes were inputted into the CMap platform, where they were filtered and ranked. Ultimately, ten compounds were identified as potential treatments for NAFLD and AIS, including lamotrigine, cinnarizine, VX-702, levamisole, lenvatinib, PHA-767491, EMD-53998, BRL-50481, venlafaxine, and kenpaullone. Their molecular structures are depicted in Fig 8.

### 3.7 External and experimental validation

**Fig 9:**
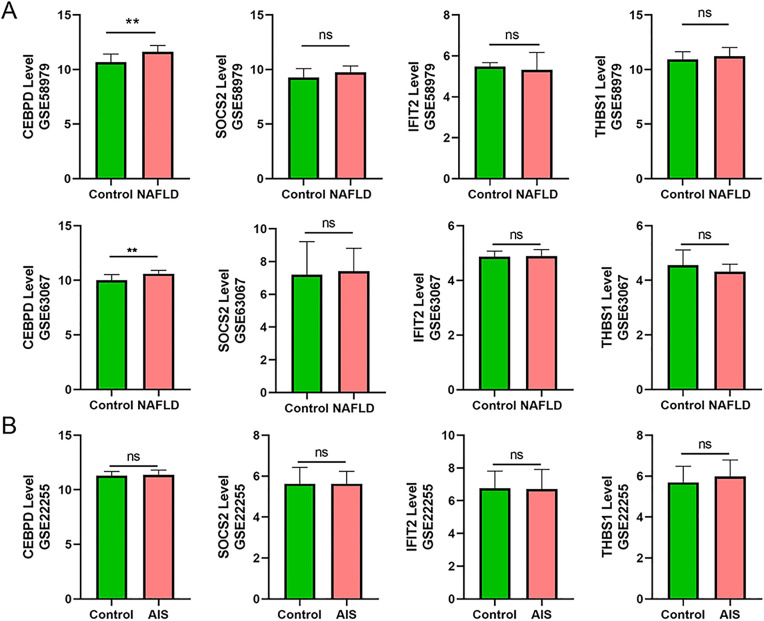
The external validation.

To validate the potential involvement of *CEBPD*, *SOCS2*, *THBS1*, and *IFIT2* in NAFLD pathogenesis, we performed comprehensive analyses using both clinical datasets and experimental models. In clinical NAFLD datasets (GSE58979 and GSE63067), *CEBPD* expression was significantly elevated in NAFLD patients compared to healthy controls (*p* < 0.05), while *SOCS2*, *THBS1*, and *IFIT2* levels showed no significant changes. Notably, in the acute ischemic stroke (AIS) dataset (GSE22255), none of these genes exhibited differential expression, suggesting their specific association with NAFLD rather than general liver injury (Fig 9A, B).

**Fig 10:**
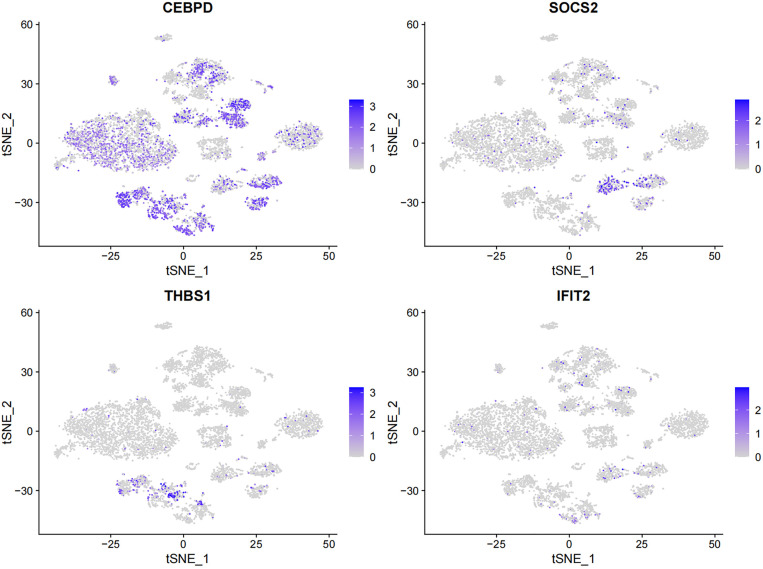
Expression patterns of four key genes (*CEBPD*, *SOCS2*, *THBS1*, and *IFIT2*) across different liver cell types.

The t-SNE plot illustrates the expression patterns of four key genes (*CEBPD*, *SOCS2*, *THBS1*, and *IFIT2*) across different cell types in liver tissue. *CEBPD* and *THBS1* were broadly expressed in multiple cell types, with particularly high expression observed in hepatocytes and endothelial cells. *SOCS2* showed more pronounced expression in B cells and T cells, whereas *IFIT2* exhibited relatively low expression, mainly restricted to subsets of immune cells such as T cells and macrophages. Notably, hepatocyte displayed the greatest heterogeneity, especially with respect to *CEBPD* and *THBS1* expression(Fig 10, S1 Fig).

**Fig 11:**
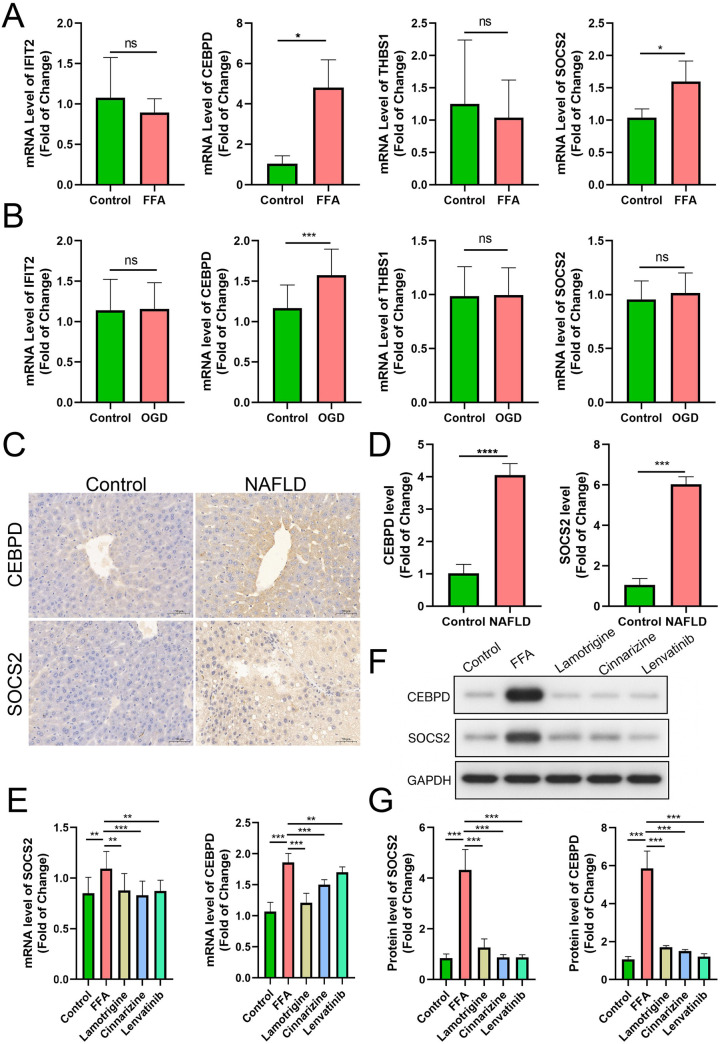
The results of experiments. (A) FFA-treated HepG2 cells. (B) OGD-induced HMC3 microglial cells. (C) IHC analysis in NAFLD mouse model. (D) Drug validation. (E) The results of qPCR. (F,G) Western blot analysis of *CEBPD* and *SOCS2* expression in control, FFA, Lamotrigine, Cinnarizine, and Lenvatinib groups, with *GAPDH* as loading control.

qPCR Validation and Western blot analysis showed that the expression levels of *CEBPD* and *SOCS2* were markedly increased in the FFA group compared with the control group, while treatment with Lamotrigine, Cinnarizine, or Lenvatinib reduced their expression (*p* < 0.05). *GAPDH* was used as the loading control (Fig 11E–G).

Consistent with clinical observations, our in vitro experiments demonstrated that FFA-treated HepG2 cells showed significantly increased expression of both *CEBPD* and *SOCS2* (*p* < 0.05), with no changes in *THBS1* or *IFIT2*. Similarly, in OGD-induced HMC3 microglial cells, only *CEBPD* expression was significantly altered (*p* < 0.05) (Fig 11A, B). The translational relevance of these findings was further confirmed in our NAFLD mouse model, where IHC analysis revealed significantly elevated protein levels of both *CEBPD* and *SOCS2* in liver tissues compared to controls (*p* < 0.01) (Fig 11C).

To further explore potential therapeutic strategies targeting these NAFLD-associated genes, we investigated the effects of lamotrigine (50 μM), cinnarizine (10 μM), and lenvatinib (100 nM) on FFA-induced HepG2 cells. Notably, all three compounds significantly attenuated the FFA-induced upregulation of both *CEBPD* and *SOCS2* (*p* < 0.05) (Fig 11D).

## 4 Discussion

NAFLD is characterized by excessive fat accumulation in the liver, accompanied by complex and varied pathological mechanisms [20]. Research has identified NAFLD as an independent risk factor for cardiovascular disease [21], and patients with NAFLD have a significantly higher likelihood of developing acute cerebral infarction compared to those without the condition [22]. As the global prevalence of NAFLD rises, the associated social pressures and economic burdens become increasingly significant. This study investigates the potential relationship and shared pathological mechanisms between NAFLD and acute ischemic stroke (AIS), aiming to establish a diagnostic and preventive model for both conditions to mitigate disease occurrence and progression.

We extracted gene expression matrices and performed differential gene screening, identifying 65 common DEGs between NAFLD and AIS. GO and KEGG pathway enrichment analyses indicated that the biological processes associated with these DEGs predominantly involve immune response-regulating cell surface receptors, lymphocyte differentiation, mononuclear cell differentiation, and immune response-activating signaling pathways. The results of the functional enrichment analysis focused on immune-related pathways, and subsequent immune infiltration analysis revealed a significant increase in monocytes, plasma cells, and B cells in the NAFLD group compared to healthy controls. This highlights the role of inflammation and immune cell differentiation in NAFLD’s pathogenesis. AIS also features sterile inflammation as a pathological characteristic, suggesting that immune inflammation regulation is crucial in both diseases.

In fact, The identification of common immune regulation and inflammatory response pathways between NAFLD and AIS is particularly significant. Both diseases involve a chronic, low-grade inflammatory response, which is central to their progression. In NAFLD, immune activation is driven by excessive lipid accumulation in the liver, leading to the activation of Kupffer cells, monocytes, and macrophages, which exacerbate liver injury and fibrosis. Similar immune mechanisms are involved in AIS, where ischemic injury to the brain triggers the activation of microglia and the recruitment of peripheral immune cells, such as monocytes, to the site of injury. These findings suggest that immune modulation could be a shared therapeutic target for both diseases. This commonality in immune pathways has been well-documented in previous studies.

The liver plays a vital role in the immune system, housing Kupffer cells, which are specialized macrophages comprising 80–90% of all tissue macrophages in the body. It also contains the highest density of natural killer (NK) cells and natural killer T (NKT) cells, along with the most extensive reticuloendothelial cell network [23]. The pathogenesis of NAFLD involves various immune cells, including those engaged in innate and adaptive immune responses. Monocytes in the blood can differentiate into macrophages in the liver, primarily Kupffer cells, contributing to liver inflammation and fat accumulation [24]. In NAFLD, monocytes differentiate into phagocytes, with monocyte-derived Kupffer cells (MoKCs) responding to embryo-derived Kupffer cells (EmKCs). These MoKCs, which are partially immature and exhibit a pro-inflammatory state, exacerbate liver damage in NAFLD [25]. In addition, B cells have been shown to impact immune-mediated inflammation in NAFLD, particularly in NASH, by promoting pro-inflammatory responses [26]. The overlap of immune-related pathways between NAFLD and AIS suggests that targeting immune pathways could offer therapeutic opportunities for both diseases. However, a limitation of this study is the use of the LM22 matrix for immune infiltration analysis, which may not fully capture the liver-specific immune landscape in NAFLD.

By combining WGCNA with various machine learning methods, we identified six key genes as biomarkers for NAFLD and AIS: *CEBPD*, *SOCS2*, *THBS1*, *IFIT2*, *TNFSF10*, and *IL2RB*. *CEBPD* is a transcription factor that regulates numerous biological processes, including cell differentiation, motility, growth arrest, proliferation, cell death, metabolism, and immune responses [27]. In NAFLD, *CEBPD* may modulate inflammatory pathways, while in neurological diseases, it has been shown to exacerbate neuronal injury. Downregulating *CEBPD* can lead to reduced neurofunctional deficits, smaller infarct sizes, improved cell survival, and decreased apoptosis and oxidative stress both in vivo and in vitro, suggesting that *CEBPD* could be a new target for stroke treatment [28].

*SOCS2* serves as an important inhibitor of cytokine signaling. In NAFLD, it functions oppositely in hepatocytes and macrophages, exacerbating hepatic steatosis through the GH signaling pathway in hepatocytes, while inhibiting inflammation in macrophages by limiting the NF-κB signaling and inflammasome pathways [29,30]. In experimental autoimmune encephalomyelitis (EAE) mouse models, *SOCS2* has been found to induce neuroinflammation during the initial and peak phases of EAE [31]. *THBS1* is involved in various physiological and pathological processes, including tumor development, fibrosis, angiogenesis, cell migration, and proliferation, and may influence necroptosis through its interaction with TAK1 [32]. Its upregulation in both NAFLD and AIS indicates its potential role in the pathological processes associated with cerebral infarction by affecting cell apoptosis.

*IFIT2* belongs to the interferon-stimulated gene (ISG) family, and given that interferons are crucial regulators of inflammatory responses, *IFIT2* may participate indirectly in these processes. *TNFSF10*, also known as *TRAIL*, is a key molecule in neurological diseases. Research on Alzheimer’s disease has shown T*NFSF10*’s association with inflammatory responses and cognitive decline, with neutralization of *TNFSF10* significantly restoring cognitive behavior and reducing inflammatory mediators in tissues [33]. This suggests that *TNFSF10* plays a significant role in apoptosis and immune regulation. In our study, the *TNFSF10* levels in the AIS group were significantly higher than those in the healthy control group, promoting inflammation and apoptosis in the brain.

However, through the validation of the datasets GSE58979 and GSE63067, we found that there was no significant difference in *SOCS2*, *THBS1* and *IFIT2* between the control and NAFLD groups, which led us to conclude that these three genes might be more related to fat metabolism.

Studies have indicated that overexpression of *SOCS2* can significantly inhibit the expression of Leptin receptor in adipocytes [34].THBS1 is an obesity-associated matricellular protein, which induces proliferation of fibro-adipogenic progenitors (FAPs) — mesenchymal cells that differentiate into adipocytes and fibroblasts. It has been shown that *IFIT2* inhibits cholesterol efflux via PPARγ/LXRα/ABCA1-ABCG1 pathway [35,36].

To morbidly obese patients, the difference of *CEBPD* between control group and NAFLD group was significant. It can be seen that the reason for this outcome may be that *SOCS2*, *THBS1* and *IFIT2* are involved in the development of NAFLD by their involvement in fat metabolism, thereby affecting the pathogenesis of diseases.Based on this, we think *CEBPD* is more valuable to our study.

Experimental results revealed that the expression level of *CEBPD* and *SOCS2* significantly increased in high-fat-induced liver cells and liver tissue of NFALD mice, indicating that *CEBPD* and *SOCS2* may play a key role in both NAFLD and AIS and could serve as a potential diagnostic marker for AIS in NAFLD patients. While *CEBPD* and *SOCS2* showed significant results in qPCR validation, the other candidate genes did not. Possible reasons include biological variability in patient samples, differences in experimental conditions, and technical noise, such as small sample sizes and PCR efficiency. These factors highlight the need for further validation in larger cohorts.

Additionally, while exploring treatments for NAFLD and AIS, we utilized the cMAP database to screen a series of potential therapeutic small molecules. Lamotrigine (LTG), a sodium channel regulator, is widely used to treat various neurological disorders, including epilepsy and bipolar disorder. It acts by blocking voltage-dependent sodium channels, reducing calcium currents, and decreasing neuronal excitability, thus inhibiting the release of neurotransmitters like glutamate and aspartate. Beyond its established mechanisms, LTG has also been shown to regulate HCN channels and modulate the activity of the inhibitory neurotransmitter GABA, helping to reduce abnormal neurotransmitter release and protect brain tissue [37–39]. While LTG is primarily used in neurological diseases, its anti-inflammatory and neuroprotective properties suggest that it could also have therapeutic potential in AIS, particularly in reducing neuronal injury and inflammation following ischemic events.

BRL-50481 is a selective phosphodiesterase 7 (PDE7) inhibitor that increases intracellular cAMP levels by inhibiting PDE7 activity, thereby regulating associated inflammatory and immune responses [40]. Additionally, BRL-50481 can prevent the deterioration of recognition memory and neuronal apoptosis by restoring cAMP levels and activating the cAMP/CREB signaling pathway in the hippocampus [41]. These drugs present new avenues for future treatments [42].

While Lamotrigine (LTG) and BRL-50481 show promise for treating NAFLD and AIS, their safety profiles in these patient populations need careful consideration. Lamotrigine is generally well tolerated in neurological patients, but in those with NAFLD or AIS, it could present risks, including liver toxicity and cardiovascular side effects such as arrhythmias. LTG’s hepatic metabolism requires monitoring, especially in NAFLD patients with liver dysfunction. BRL-50481, a PDE7 inhibitor, has shown potential in neuronal protection, but its impact on metabolic processes and vascular tone remains unclear, particularly in patients with metabolic syndrome or vascular risk.

Our experimental results showed that lamotrigine, cinnarizine, and lenvatinib significantly suppressed the expression of *CEBPD* and *SOCS2* in FFA-induced HepG2 cells, supporting their potential roles in regulating hepatic inflammation. Lamotrigine may exert protective effects through its anti-inflammatory properties, while cinnarizine and lenvatinib may act through calcium signaling and kinase inhibition pathways. However, a limitation of this study is the use of HepG2 cells, a hepatocellular carcinoma line, which may not fully recapitulate normal hepatocyte physiology. To address this, future studies will validate these findings in primary hepatocytes and animal models to better assess the mechanistic roles, safety, and therapeutic potential of the identified compounds in liver disease.

### Conclusion

This study identified shared molecular features between NAFLD and AIS through integrative transcriptomic analyses, highlighting *CEBPD* and *SOCS2* as key genes specifically associated with NAFLD. These findings were consistently validated across clinical datasets, in vitro cell models, and in vivo mouse experiments. Furthermore, pharmacological intervention using lamotrigine, cinnarizine, and lenvatinib effectively downregulated *CEBPD* and SOCS2 expression in FFA-induced hepatocyte models, suggesting potential therapeutic relevance. Collectively, our results provide novel insights into the molecular mechanisms underlying NAFLD and offer promising targets for future drug development.

## Supporting information

S1 Fig Bubble chart showing the expression levels of Lasso genes across different liver tissue cell clusters. (PDF)

S1 Table (A) Potential transcription factor for CEBPD. (B) Potential transcription factor for SOCS2.
(PDF)

S1 File Packages and R code used for data validation. (PDF)

## References

[1] Younossi ZM, Zelber-Sagi S, Henry L, Gerber LH. Lifestyle interventions in nonalcoholic fatty liver disease. Nat Rev Gastroenterol Hepatol. 2023;20(11):708–22. doi: 10.1038/s41575-023-00800-4 37402873

[2] Rong L, Zou J, Ran W, Qi X, Chen Y, Cui H, et al. Advancements in the treatment of non-alcoholic fatty liver disease (NAFLD). Front Endocrinol (Lausanne). 2023;13:1087260. doi: 10.3389/fendo.2022.1087260 36726464 PMC9884828

[3] Wen W, Wu P, Zhang Y, Chen Z, Sun J, Chen H. Comprehensive analysis of NAFLD and the therapeutic target identified. Front Cell Dev Biol. 2021;9:704704. doi: 10.3389/fcell.2021.704704 34616724 PMC8488166

[4] Parikh NS. Mendelian randomization elucidates links between nonalcoholic fatty liver disease and stroke. Eur J Neurol. 2022;29(5):1291–2. doi: 10.1111/ene.15313 35263808

[5] Xu J, Dai L, Zhang Y, Wang A, Li H, Wang Y. Severity of nonalcoholic fatty liver disease and risk of future ischemic stroke events. Stroke. 2021;52(1):103–10. 33272125 10.1161/STROKEAHA.120.030433

[6] Putaala J. Ischemic stroke in young adults. Continuum (Minneap Minn). 2020;26(2):386–414. doi: 10.1212/CON.0000000000000833 32224758

[7] Feske SK. Ischemic stroke. Am J Med. 2021;134(12):1457–64. 34454905 10.1016/j.amjmed.2021.07.027

[8] GBD 2019 Stroke Collaborators. Global, regional, and national burden of stroke and its risk factors, 1990-2019: a systematic analysis for the Global Burden of Disease Study 2019. Lancet Neurol. 2021;20(10):795–820. 34487721 10.1016/S1474-4422(21)00252-0 PMC8443449

[9] Potter TBH, Tannous J, Vahidy FS. A contemporary review of epidemiology, risk factors, etiology, and outcomes of premature stroke. Curr Atheroscler Rep. 2022;24(12):939–48. doi: 10.1007/s11883-022-01067-x 36374365 PMC9660017

[10] Yan M, Man S, Sun B, Ma L, Guo L, Huang L, et al. Gut liver brain axis in diseases: the implications for therapeutic interventions. Signal Transduct Target Ther. 2023;8(1):443. doi: 10.1038/s41392-023-01673-4 38057297 PMC10700720

[11] Xu K, Lu Y, Hou S, Liu K, Du Y, Huang M, et al. Detecting anomalous anatomic regions in spatial transcriptomics with STANDS. Nat Commun. 2024;15(1):8223. doi: 10.1038/s41467-024-52445-9 39300113 PMC11413068

[12] Arendt BM, Comelli EM, Ma DWL, Lou W, Teterina A, Kim T, et al. Altered hepatic gene expression in nonalcoholic fatty liver disease is associated with lower hepatic n-3 and n-6 polyunsaturated fatty acids. Hepatology. 2015;61(5):1565–78. doi: 10.1002/hep.27695 25581263

[13] Barr TL, Conley Y, Ding J, Dillman A, Warach S, Singleton A. Genomic biomarkers and cellular pathways of ischemic stroke by RNA gene expression profiling. Neurology. 2010;75(11):1009–14. 20837969 10.1212/WNL.0b013e3181f2b37f PMC2942033

[14] du Plessis J, van Pelt J, Korf H, Mathieu C, van der Schueren B, Lannoo M, et al. Association of adipose tissue inflammation with histologic severity of nonalcoholic fatty liver disease. Gastroenterology. 2015;149(3):635–48.e14. doi: 10.1053/j.gastro.2015.05.044 26028579

[15] Subramanian A, Tamayo P, Mootha VK, Mukherjee S, Ebert BL, Gillette MA, et al. Gene set enrichment analysis: a knowledge-based approach for interpreting genome-wide expression profiles. Proc Natl Acad Sci U S A. 2005;102(43):15545–50. doi: 10.1073/pnas.0506580102 16199517 PMC1239896

[16] Deng T, Chen S, Zhang Y, Xu Y, Feng D, Wu H. A cofunctional grouping-based approach for non-redundant feature gene selection in unannotated single-cell RNA-seq analysis. Brief Bioinform. 2023;24(2):bbad042. doi: 10.1093/bib/bbad042 PMC10025445 36754847

[17] Zhang J-J, Shen Y, Chen X-Y, Jiang M-L, Yuan F-H, Xie S-L, et al. Integrative network-based analysis on multiple Gene Expression Omnibus datasets identifies novel immune molecular markers implicated in non-alcoholic steatohepatitis. Front Endocrinol (Lausanne). 2023;14:1115890. doi: 10.3389/fendo.2023.1115890 37008925 PMC10061151

[18] Mahmoudi A, Butler AE, Majeed M, Banach M, Sahebkar A. Investigation of the effect of curcumin on protein targets in NAFLD using bioinformatic analysis. Nutrients. 2022;14(7):1331. doi: 10.3390/nu14071331 35405942 PMC9002953

[19] Dong G, Gao H, Chen Y, Yang H. Machine learning and bioinformatics analysis to identify autophagy-related biomarkers in peripheral blood for rheumatoid arthritis. Front Genet. 2023;14:1238407. doi: 10.3389/fgene.2023.1238407 37779906 PMC10533932

[20] Alisi A, Marcellini M, Nobili V. Bioinformatics as tool to identify gene/protein-pathways associated with nonalcoholic fatty liver disease/nonalcoholic steatohepatitis. Hepatology. 2007;46(4):1306; author reply 1306-7. doi: 10.1002/hep.21910 17894316

[21] Targher G, Byrne CD, Tilg H. NAFLD and increased risk of cardiovascular disease: clinical associations, pathophysiological mechanisms and pharmacological implications. Gut. 2020;69(9):1691–705. doi: 10.1136/gutjnl-2020-320622 32321858

[22] Cao Y, Du Y, Jia W, Ding J, Yuan J, Zhang H, et al. Identification of biomarkers for the diagnosis of chronic kidney disease (CKD) with non-alcoholic fatty liver disease (NAFLD) by bioinformatics analysis and machine learning. Front Endocrinol (Lausanne). 2023;14:1125829. doi: 10.3389/fendo.2023.1125829 36923221 PMC10009268

[23] Jenne CN, Kubes P. Immune surveillance by the liver. Nat Immunol. 2013;14(10):996–1006. doi: 10.1038/ni.2691 24048121

[24] Miceli C, Leri M, Stefani M, Bucciantini M. Autophagy-related proteins: potential diagnostic and prognostic biomarkers of aging-related diseases. Ageing Res Rev. 2023;89:101967. doi: 10.1016/j.arr.2023.101967 37270146

[25] Tran S, Baba I, Poupel L, Dussaud S, Moreau M, Gélineau A. Impaired kupffer cell self-renewal alters the liver response to lipid overload during non-alcoholic steatohepatitis. Immunity. 2020;53(3):627–40.e5. doi: 10.1016/j.immuni.2020.08.008 32562600

[26] Huby T, Gautier EL. Immune cell-mediated features of non-alcoholic steatohepatitis. Nat Rev Immunol. 2022;22(7):429–43. doi: 10.1038/s41577-021-00639-3 34741169 PMC8570243

[27] Ko C-Y, Chang W-C, Wang J-M. Biological roles of CCAAT/Enhancer-binding protein delta during inflammation. J Biomed Sci. 2015;22(1):6. doi: 10.1186/s12929-014-0110-2 25591788 PMC4318212

[28] Chen N, Xu Y, Liu Y, Zhao H, Liu R, Zhang Z. CEBPD aggravates apoptosis and oxidative stress of neuron after ischemic stroke by Nrf2/HO-1 pathway. Exp Cell Res. 2024;440(1):114127. doi: 10.1016/j.yexcr.2024.114127 38857839

[29] Zhang Z, Wang S, Zhu Z, Nie B. Identification of potential feature genes in non-alcoholic fatty liver disease using bioinformatics analysis and machine learning strategies. Comput Biol Med. 2023;157:106724. 36898287 10.1016/j.compbiomed.2023.106724

[30] Li S, Han S, Jin K, Yu T, Chen H, Zhou X, et al. SOCS2 suppresses inflammation and apoptosis during NASH progression through limiting NF-κB activation in macrophages. Int J Biol Sci. 2021;17(15):4165–75. doi: 10.7150/ijbs.63889 34803490 PMC8579457

[31] Cramer A, de Lima Oliveira BC, Leite PG, Rodrigues DH, Brant F, Esper L, et al. Role of SOCS2 in the regulation of immune response and development of the experimental autoimmune encephalomyelitis. Mediators Inflamm. 2019;2019:1872593. doi: 10.1155/2019/1872593 31949423 PMC6942913

[32] Hu H, Ma J, Peng Y, Feng R, Luo C, Zhang M, et al. Thrombospondin-1 regulates trophoblast necroptosis via NEDD4-mediated ubiquitination of TAK1 in preeclampsia. Adv Sci (Weinh). 2024;11(21):e2309002. doi: 10.1002/advs.202309002 38569496 PMC11151050

[33] Cantarella G, Di Benedetto G, Puzzo D, Privitera L, Loreto C, Saccone S, et al. Neutralization of TNFSF10 ameliorates functional outcome in a murine model of Alzheimer’s disease. Brain. 2015;138(Pt 1):203–16. doi: 10.1093/brain/awu318 25472798 PMC4441080

[34] Zhang T, Chen Y, Cai J, Pan M, Sun Q, Zhang J. SOCS2 inhibits mitochondrial fatty acid oxidation via suppressing LepR/JAK2/AMPK signaling pathway in mouse adipocytes. Oxid Med Cell Longev. 2020;2020:3742542. 32733634 10.1155/2020/3742542 PMC7376435

[35] Chen H, Wu H, Wang Q, Zhang H. IFIT2 mediates iron retention and cholesterol efflux in atherosclerosis. Int Immunopharmacol. 2024;142:113131. 39276454 10.1016/j.intimp.2024.113131

[36] Buras ED, Woo M-S, Kaul Verma R, Kondisetti SH, Davis CS, Claflin DR, et al. Thrombospondin-1 promotes fibro-adipogenic stromal expansion and contractile dysfunction of the diaphragm in obesity. JCI Insight. 2024;9(16):e175047. doi: 10.1172/jci.insight.175047 38954467 PMC11343600

[37] Costa B, Vale N. Understanding Lamotrigine’s role in the CNS and possible future evolution. Int J Mol Sci. 2023;24(7):6050. doi: 10.3390/ijms24076050 37047022 PMC10093959

[38] Cyrkler M, Drabik A, Czerwiak KZ, Soroka E. Lamotrigine: a safe and effective mood stabilizer for bipolar disorder in reproductive-age adults. Med Sci Monit. 2024;30:e945464. 10.12659/MSM.945464 PMC11468148 39370636

[39] de Miranda AS, de Miranda AS, Teixeira AL. Lamotrigine as a mood stabilizer: insights from the pre-clinical evidence. Expert Opin Drug Discov. 2019;14(2):179–90. doi: 10.1080/17460441.2019.1553951 30523725

[40] Smith SJ, Cieslinski LB, Newton R, Donnelly LE, Fenwick PS, Nicholson AG. Discovery of BRL 50481 [3-(N,N-dimethylsulfonamido)-4-methyl-nitrobenzene], a selective inhibitor of phosphodiesterase 7: in vitro studies in human monocytes, lung macrophages, and CD8 T-lymphocytes. Mol Pharmacol. 2004;66(6):1679–89. 15371556 10.1124/mol.104.002246

[41] Chen Y, Li S, Zhong X, Kang Z, Chen R. PDE-7 inhibitor BRL-50481 reduces neurodegeneration and long-term memory deficits in mice following sevoflurane exposure. ACS Chem Neurosci. 2020;11(9). 10.1021/acschemneuro.0c00106 32271540

[42] Chen L, Wang L, Ao W, Chen Y, Li S, Huang Z, et al. Bioinformatics study of the potential therapeutic effects of ginsenoside Rf in reversing nonalcoholic fatty liver disease. Biomed Pharmacother. 2022;149:112879. doi: 10.1016/j.biopha.2022.112879 35358801

