# Supplementary figures and images for: Uncovering biomarkers and molecular pathways linking NAFLD and AIS: Insights from bioinformatic analysis and experiment
Source: PLoS One. 2025 Sep 29;20(9):e0333719. doi: 10.1371/journal.pone.0333719 (PMC12478928; doi:10.1371/journal.pone.0333719)

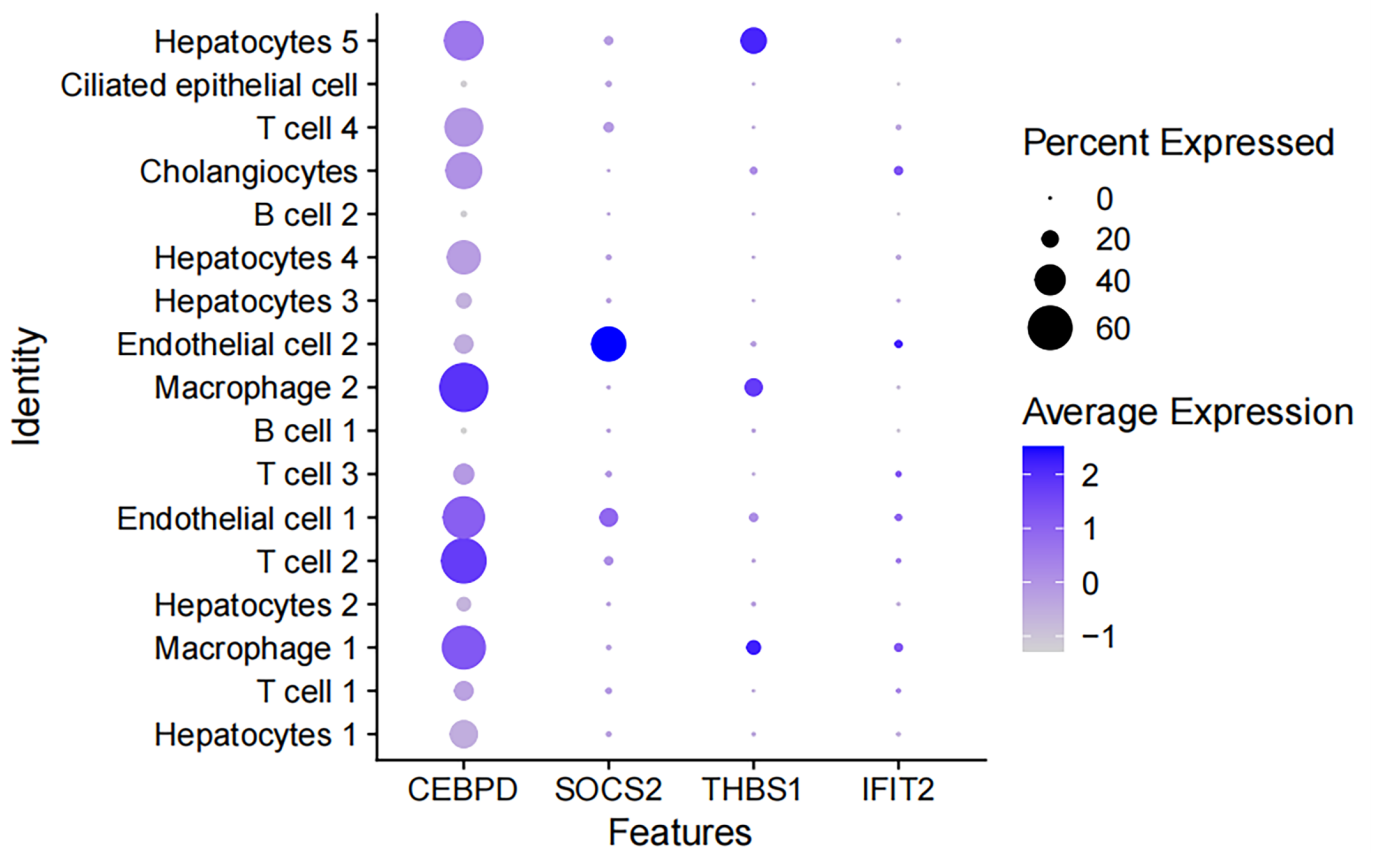

Supplement: S1 Fig — (PDF) [file pone.0333719.s001.pdf]
