# Supplementary material for: Uncovering biomarkers and molecular pathways linking NAFLD and AIS: Insights from bioinformatic analysis and experiment
Source: PLoS One. 2025 Sep 29;20(9):e0333719. doi: 10.1371/journal.pone.0333719 (PMC12478928; doi:10.1371/journal.pone.0333719)
Supplement: S1 Table — (B) Potential transcription factor for SOCS2. (PDF) [file pone.0333719.s002.pdf]

S1A Table. Potential transcription factor for CEBPD

TF	Tissue	The peak close to TSS	The peak with strongest signal
CREB1	liver	chr8,47738398,47738687,4.26,399,pr,dataset-4388	chr8,47738084,47738268,27.9,818,gb,dataset-4388
CTCF	liver	chr8,47738999,47739162,37.5,-76,pr,dataset-747	chr8,47738999,47739162,37.5,-76,pr,dataset-747
ELF1	liver	chr8,47738943,47739135,4.60,-49,pr,dataset-857	chr8,47738371,47738600,13.0,486,pr,dataset-857
FOXA1	liver	chr8,47738886,47739089,3.80,-3,pr,dataset-1274	chr8,47738886,47739089,3.80,-3,pr,dataset-1274
FOXA2	liver	chr8,47737581,47739119,13.1,-33,pr,dataset-5056	chr8,47737581,47739119,15.2,-33,pr,dataset-5056
HDAC2	liver	chr8,47738812,47739121,4.37,-35,pr,dataset-1591	chr8,47738031,47738178,5.36,908,gb,dataset-1591
JUND	liver	chr8,47739875,47740100,12.5,-101,pr,dataset-1826	chr8,47739875,47740100,12.5,-101,pr,dataset-1826
MAX	liver	chr8,47738862,47739051,2.89,35,pr,dataset-1972	chr8,47737984,47738220,10.1,866,gb,dataset-1964
MAZ	liver	chr8,47738993,47739114,4.34,-28,pr,dataset-1989	chr8,47738324,47738545,4.54,541,gb,dataset-1989
MXI1	liver	chr8,47738949,47739109,5.76,-23,pr,dataset-2067	chr8,47738949,47739109,5.76,-23,pr,dataset-2067
POLR2A	liver	chr8,47738881,47739042,6.18,44,pr,dataset-2501	chr8,47737882,47738255,30.4,831,gb,dataset-2501
RAD21	liver	chr8,47738882,47739185,24.7,-99,pr,dataset-2616	chr8,47738802,47739313,31.3,-227,pr,dataset-2601
SMC3	liver	chr8,47738992,47739307,20.9,-221,pr,dataset-3009	chr8,47738992,47739307,20.9,-221,pr,dataset-3009
STAG1	liver	chr8,47738793,47739218,48.2,-132,pr,dataset-3354	chr8,47738793,47739218,48.2,-132,pr,dataset-3354
TAF1	liver	chr8,47738039,47738222,4.37,864,gb,dataset-3472	chr8,47737619,47737774,4.84,1312,gb,dataset-3472
YY1	liver	chr8,47738490,47739172,7.69,-86,pr,dataset-3765	chr8,47737266,47738300,25.3,786,gb,dataset-3765
ZBTB7A	liver	chr8,47738975,47739108,5.50,-22,pr,dataset-3797	chr8,47738062,47738428,8.62,658,gb,dataset-3797

S1B Table. Potential transcription factor for SOCS2

TF	Tissue	The peak close to TSS	The peak with strongest signal
BHLHE40	liver	chr12,93569673,93569916,4.11,-141,pr,dataset-235	chr12,93570555,93571361,50.1,741,gb,dataset-235
CEBPA	liver	chr12,93569773,93569982,18.7,-41,pr,dataset-408	chr12,93563898,93564132,25.4,-591,pr,dataset-408
CEBPB	liver	chr12,93569777,93569918,12.3,-37,pr,dataset-417	chr12,93569727,93569987,16.7,-87,pr,dataset-421
CEBPD	liver	chr12,93569753,93570113,4.62,-61,pr,dataset-438	chr12,93570843,93570958,5.06,1029,gb,dataset-438
CREB1	liver	chr12,93569721,93569855,10.6,-93,pr,dataset-4390	chr12,93563834,93564179,49.7,-598,pr,dataset-4388
CTCF	liver	chr12,93569663,93569803,16.0,-151,pr,dataset-747	chr12,93569648,93569896,41.6,-166,pr,dataset-575
ELF1	liver	chr12,93569703,93569902,7.33,-111,pr,dataset-857	chr12,93570834,93571012,7.57,1020,gb,dataset-857
EP300	liver	chr12,93569693,93570002,8.80,-121,pr,dataset-905	chr12,93563766,93564228,23.9,-604,pr,dataset-905
FOXA1	liver	chr12,93569683,93569913,5.40,-131,pr,dataset-1274	chr12,93563751,93564225,21.5,-606,pr,dataset-1274
FOXA2	liver	chr12,93569699,93569903,5.60,-115,pr,dataset-1345	chr12,93563740,93564200,15.4,-607,pr,dataset-1345
GABPA	liver	chr12,93569632,93569963,15.9,-182,pr,dataset-4387	chr12,93563760,93564186,34.5,-605,pr,dataset-4387
HDAC2	liver	chr12,93569670,93570250,7.77,-144,pr,dataset-1591	chr12,93563741,93564239,15.7,-607,pr,dataset-1591
JUN	liver	chr12,93569833,93569973,4.93,19,pr,dataset-1808	chr12,93563855,93564211,6.99,-595,pr,dataset-1808
MAX	liver	chr12,93570711,93571381,7.73,897,gb,dataset-1972	chr12,93570788,93571415,13.4,974,gb,dataset-1964
MAZ	liver	chr12,93569643,93570016,6.12,-171,pr,dataset-1989	chr12,93570854,93571074,7.82,1040,gb,dataset-1989
NFE2	liver	chr12,93569633,93569937,5.65,-181,pr,dataset-2227	chr12,93570692,93571098,8.94,878,gb,dataset-2227
NR2F2	liver	chr12,93569760,93569929,3.19,-54,pr,dataset-2309	chr12,93563863,93564210,8.77,-595,pr,dataset-2309
POLR2A	liver	chr12,93570014,93570146,4.55,200,pr,dataset-2524	chr12,93570724,93571109,6.55,910,gb,dataset-2524
RAD21	liver	chr12,93569641,93569905,18.6,-173,pr,dataset-2616	chr12,93569548,93569991,52.2,-266,pr,dataset-2601
RXRA	liver	chr12,93569643,93570002,15.8,-171,pr,dataset-2865	chr12,93570803,93571045,16.2,989,gb,dataset-2865

SIN3A	liver	chr12,93569718,93569871,5.21,-96,pr,dataset-2902	chr12,93569718,93569871,5.21,-96,pr,dataset-2902
SMC3	liver	chr12,93569620,93570030,15.2,-194,pr,dataset-3009	chr12,93569620,93570030,15.2,-194,pr,dataset-3009
STAG1	liver	chr12,93569629,93569940,45.1,-185,pr,dataset-3354	chr12,93569629,93569940,45.1,-185,pr,dataset-3354
SUZ12	liver	chr12,93570824,93570994,5.31,1010,gb,dataset-3462	chr12,93570824,93570994,5.31,1010,gb,dataset-3462
USF1	liver	chr12,93570767,93570998,56.8,953,gb,dataset-3705	chr12,93570767,93570998,56.8,953,gb,dataset-3705
USF2	liver	chr12,93570770,93570998,13.7,956,gb,dataset-3716	chr12,93570770,93570998,13.7,956,gb,dataset-3716
YY1	liver	chr12,93569658,93569891,10.4,-156,pr,dataset-3765	chr12,93569658,93569891,10.4,-156,pr,dataset-3765
