# Supplementary material for: Uncovering biomarkers and molecular pathways linking NAFLD and AIS: Insights from bioinformatic analysis and experiment
Source: PLoS One. 2025 Sep 29;20(9):e0333719. doi: 10.1371/journal.pone.0333719 (PMC12478928; doi:10.1371/journal.pone.0333719)
Supplement: S1 File — (PDF) [file pone.0333719.s003.pdf]

S1 File.Packages and R code used for data validation:

1.GEO Data Compilation

```
library(tidyverse)
library(limma)
setwd("GSE89632")
exp <- read.table("GSE89632_series_matrix.txt",header=T,
                  sep="\t",dec=".",
                  comment.char="!",na.strings =c("NA"),fill=T )
plate <- read.table("GPL14951-11332.txt",header=T,
                    quote="",sep="\t",dec=".",
                    comment.char="#",na.strings =c("NA"),fill=T )
2.Data standardisation processing
ID <- data.frame(ID_REF = plate$ID, Gene_Symbol = plate$Symbol)
ID$Gene_Symbol
<-data.frame(sapply(ID$Gene_Symbol,function(x)unlist(strsplit(x,"///"))[1]),stringsAsFactors=F)[,1]
exp <- as.data.frame(exp)
exp <- merge(exp, ID, by='ID_REF')#merge()
exp[, grep("Gene_Symbol", colnames(exp))] <-    trimws(exp[, grep("Gene_Symbol", colnames(exp))])
exp[exp == ""]
exp <- na.omit(exp)
table(duplicated(exp$Gene_Symbol))
exp1 <- avereps(exp, ID = exp$Gene_Symbol)
exp1 <- as.data.frame(exp1)
rownames(exp1) <- exp1$Gene_Symbol
exp1 <- exp1[,-c(1,ncol(exp1))]
write.csv(exp1, "exp_average.csv")
```

2.Extract differentially expressed genes and generate gene heatmaps and volcano plots

```
library(limma)
library(pheatmap)
library(ggplot2)
library(ggrepel)
inputFile="exp_average.csv"
conFile="s1.txt"
treatFile="s2.txt"
logFCfilter=0.585
adj.P.Val.Filter=0.05
dimnames=list(rownames(exp),colnames(exp))
data=matrix(as.numeric(as.matrix(exp)),nrow=nrow(exp),dimnames=dimnames)
rt=avereps(data)

qx=as.numeric(quantile(rt, c(0, 0.25, 0.5, 0.75, 0.99, 1.0), na.rm=T))
LogC=( (qx[5]>100) || ( (qx[6]-qx[1])>50 && qx[2]>0) )
```

```
if(LogC){
   rt[rt<0]=0
   rt=log2(rt+1)}
data=normalizeBetweenArrays(rt)

sample1=read.table(conFile, header=F, sep="\t", check.names=F)
sample2=read.table(treatFile, header=F, sep="\t", check.names=F)
sampleName1=gsub("^ | $", "", as.vector(sample1[,1]))
sampleName2=gsub("^ | $", "", as.vector(sample2[,1]))
conData=data[,sampleName1]
treatData=data[,sampleName2]
data=cbind(conData,treatData)
conNum=ncol(conData)
treatNum=ncol(treatData)

Type=c(rep("Control",conNum), rep("NAFLD",treatNum))
design <- model.matrix(~0+factor(Type))
colnames(design) <- c("NAFLD","Control")
fit <- lmFit(data,design)
cont.matrix<-makeContrasts(NAFLD-Control,levels=design)
fit2 <- contrasts.fit(fit, cont.matrix)
fit2 <- eBayes(fit2)

allDiff=topTable(fit2, adjust='fdr', number=200000)
allDiffOut <- allDiff
write.table(allDiffOut, file="all.txt", sep="\t", quote=F, col.names=T)

Type=c(rep("Control",conNum),rep("NAFLD",treatNum))
outData=rbind(id=paste0(colnames(data),"_",Type),data)
write.table(outData, file="normalize.txt", sep="\t", quote=F, col.names=F)

diffSig=allDiff[with(allDiff, (abs(logFC)>logFCfilter & P.Value < adj.P.Val.Filter )), ]
diffSigOut=rbind(id=colnames(diffSig),diffSig)
write.table(diffSigOut,file="diff.txt",sep="\t",quote=F,col.names=F)

geneNum=50
diffSig=diffSig[order(as.numeric(as.vector(diffSig$logFC))),]
diffGeneName=as.vector(rownames(diffSig))
diffLength=length(diffGeneName)
hmGene=c()
if(diffLength>(2*geneNum)){
   hmGene=diffGeneName[c(1:geneNum,(diffLength-geneNum+1):diffLength)]
}else{
   hmGene=diffGeneName
```

```
}
hmExp=data[hmGene,]
Type=c(rep("Control",conNum),rep("NAFLD",treatNum))
names(Type)=colnames(data)
Type=as.data.frame(Type)
pdf(file="Top-50heatmap.pdf", width=9, height=6.5)
pheatmap(hmExp,
         annotation=Type,
         color = colorRampPalette(c("blue", "white", "red"))(50),
         cluster_cols =F,
         show_colnames = F,
         scale="row",
         fontsize = 7,
         fontsize_row=5,
         fontsize_col=7)
dev.off()

allDiff$logFC[allDiff$logFC>20]=20
allDiff$logFC[allDiff$logFC< -20]=-20
Significant=ifelse((allDiff$P.Value<adj.P.Val.Filter
& abs(allDiff$logFC)>logFCfilter), ifelse(allDiff$logFC>logFCfilter,"Up","Down"), "Not")

diffSig=allDiff[with(allDiff, (abs(logFC)>logFCfilter & adj.P.Val < adj.P.Val.Filter )), ]
diffSigOut=rbind(id=colnames(diffSig),diffSig)
write.table(diffSigOut,file="diff.txt",sep="\t",quote=F,col.names=F)
geneNum=10
diffSig=diffSig[order(as.numeric(as.vector(diffSig$logFC))),]
diffGeneName=as.vector(rownames(diffSig))
diffLength=length(diffGeneName)
hmGene=c()
if(diffLength>(2*geneNum)){
  hmGene=diffGeneName[c(1:geneNum,(diffLength-geneNum+1):diffLength)]
}else{hmGene=diffGeneName}
hmExp=data[hmGene,]
hmExp <- as.data.frame(hmExp)
top5.genes <- c(as.character(rownames(hmExp)))

allDiff2 <- allDiff
allDiff2$Label = ""
allDiff2$Gene <- rownames(allDiff2)
allDiff2$Label[match(top5.genes, allDiff2$Gene)] <- top5.genes
p = ggplot(allDiff2, aes(logFC, -log10(adj.P.Val)))+
  geom_point(aes(col=Significant))+
  scale_color_manual(values=c("green", "black", "red"))+
```

```
    labs(title = " ")+
    theme(plot.title = element_text(size = 16, hjust = 0.5, face = "bold"))+
    geom_hline(yintercept = -log10(0.05), linetype = "dashed") +
    geom_vline(xintercept = c(-0.5, 0.5), linetype = "dashed")+
    geom_label_repel(data = allDiff2,aes(logFC, -log10(adj.P.Val), label = Label),size = 3)
p=p+theme_bw()
pdf(file="Top10-vol2.pdf", width=10.5, height=6)
print(p)
dev.off()
```

3.GO

```
library(clusterProfiler)
library(org.Hs.eg.db)
library(enrichplot)
library(ggplot2)
library(circlize)
library(RColorBrewer)
library(dplyr)
library(ComplexHeatmap)

pvalueFilter=0.05
adjPvalFilter=1
colorSel="p.adjust"
if(adjPvalFilter>0.05){
    colorSel="pvalue"
}

setwd("/GO")
rt=read.table("diff.txt", header=F, sep="\t", check.names=F)
genes=unique(as.vector(rt[,1]))
entrezIDs=mget(genes, org.Hs.egSYMBOL2EG, ifnotfound=NA)
entrezIDs=as.character(entrezIDs)
rt=cbind(rt, entrezIDs)
rt=rt[rt[,"entrezIDs"]!="NA",]
gene=rt$entrezID

kk=enrichGO(gene=gene, OrgDb=org.Hs.eg.db, pvalueCutoff=1, qvalueCutoff=1, ont="all", readable=T)
GO=as.data.frame(kk)
GO=GO[(GO$pvalue<pvalueFilter & GO$p.adjust<adjPvalFilter),]
write.table(GO, file="GO.txt", sep="\t", quote=F, row.names = F)
pdf(file="GObarplot.pdf", width=8, height=15.8)
bar=barplot(kk, drop=TRUE, showCategory=10, label_format=30, split="ONTOLOGY", color=colorSel) +
facet_grid(ONTOLOGY~., scale='free')
print(bar)
```

```
dev.off()
```

4.KEGG

```
setwd("/kegg")
library(GOplot)
library(clusterProfiler)
library(org.Hs.eg.db)
library(enrichplot)
library(ggplot2)
library(circlize)
library(RColorBrewer)
library(dplyr)
library(ComplexHeatmap)
rt=read.table("diff.txt",sep="\t",check.names=F,header=T)
genes=as.vector(rt[,1])
entrezIDs=mget(genes, org.Hs.egSYMBOL2EG, ifnotfound=NA)
entrezIDs <- as.character(entrezIDs)
out=cbind(rt,entrezID=entrezIDs)
write.table(out,file="KEGG-id.txt",sep="\t",quote=F,row.names=F)


rt=read.table("KEGG-id.txt",sep="\t",header=T,check.names=F)
rt=rt[is.na(rt[,"entrezID"])==F,]
gene=rt$entrezID
kk <- enrichKEGG(gene = gene,keyType = "kegg",organism = "hsa", pvalueCutoff =1, qvalueCutoff =1, pAdjustMethod = "fdr")
write.table(kk,file="KEGG.txt",sep="\t",quote=F,row.names = F)

pdf(file="KEGG-barplot.pdf",width = 10,height = 13)
barplot(kk, drop = TRUE, showCategory = 15,label_format=100)
dev.off()
```

5.GSEA

```
library(limma)
library(org.Hs.eg.db)
library(clusterProfiler)
library(enrichplot)

inputFile="all.txt"
gmtFile="c2.cp.kegg.Hs.symbols.gmt"
gmtFile="c5.go.Hs.symbols.gmt"
setwd("GSEA")

rt=read.table(inputFile, header=T,row.names=NULL, sep="\t", check.names=F)
```

```
logFC=as.vector(rt[,2])
names(logFC)=as.vector(rt[,1])
logFC=sort(logFC, decreasing=T)
gmt=read.gmt(gmtFile)

kk=GSEA(logFC, TERM2GENE=gmt, pvalueCutoff = 1)
kkTab=as.data.frame(kk)
kkTab=kkTab[kkTab$p.adjust<0.05,]
write.table(kkTab,file="GSEA_KEGG.result.txt",sep="\t",quote=F,row.names = F)

termNum=5
kkUp=kkTab[kkTab$NES>0,]
if(nrow(kkUp)>=termNum){
  showTerm=row.names(kkUp)[1:termNum]
  gseaplot=gseaplot2(kk, showTerm, base_size=0, title="Enriched in NAFLD")
  pdf(file="GSEA_KEGG.pdf", width=12.5, height=8)
  print(gseaplot)
  dev.off()
}

termNum=5
kkDown=kkTab[kkTab$NES<0,]
if(nrow(kkDown)>=termNum){
  showTerm=row.names(kkDown)[1:termNum]
  gseaplot=gseaplot2(kk, showTerm, base_size=0, title="Enriched in Control")
  pdf(file="GSEA_KEGG.Control.pdf", width=12.5, height=8)
  print(gseaplot)
  dev.off()
}
```

6.WGCNA

```
setwd("/WGCNA")
library(limma)
library(pheatmap)
library(ggplot2)
library(ggrepel)
library(tidyverse)
#inputFile="exp_average.csv"
conFile="s1.txt"
treatFile="s2.txt"
logFCfilter=0.585
adj.P.Val.Filter=0.05
dimnames=list(rownames(exp),colnames(exp))
data=matrix(as.numeric(as.matrix(exp)),nrow=nrow(exp),dimnames=dimnames)
```

```
rt=avereps(data)

qx=as.numeric(quantile(rt, c(0, 0.25, 0.5, 0.75, 0.99, 1.0), na.rm=T))
LogC=( (qx[5]>100) || ( (qx[6]-qx[1])>50 && qx[2]>0) )
if(LogC){
  rt[rt<0]=0
  rt=log2(rt+1)}
data=normalizeBetweenArrays(rt)

sample1=read.table(conFile, header=F, sep="\t", check.names=F)
sample2=read.table(treatFile, header=F, sep="\t", check.names=F)
sampleName1=gsub("^ | $", "", as.vector(sample1[,1]))
sampleName2=gsub("^ | $", "", as.vector(sample2[,1]))
conData=data[,sampleName1]
treatData=data[,sampleName2]
data=cbind(conData,treatData)
conNum=ncol(conData)
treatNum=ncol(treatData)
dataend <- as.data.frame(data)
group_list <- c(rep("Control",conNum),rep("NAFLD",treatNum))
save(dataend,group_list,file = "step1-outputdata.Rdata")

library(WGCNA)
rm(list = ls())
getwd()
dir.create("./2.data quality")
setwd("2.data quality/")

load("../step1-outputdata.Rdata")
exp <- dataend
exp[1:4,1:4]
data <- exp

keep_data <- data[order(apply(data,1,mad), decreasing = T)[1:5000],]
datTraits <- data.frame(row.names = colnames(data),group=group_list)
datTraits$groupNO= ifelse(datTraits$group=="Control",'1','2')
datTraits<-as.data.frame(datTraits)

datExpr0 <- as.data.frame(t(keep_data))
datExpr0[1:4,1:4]
datTraits[1:4,1:2]


gsg <- goodSamplesGenes(datExpr0,verbose = 3)
```

```
gsg$allOK
if (!gsg$allOK){
  # Optionally, print the gene and sample names that were removed:
  if (sum(!gsg$goodGenes)>0)
    printFlush(paste("Removing genes:", paste(names(datExpr0)[!gsg$goodGenes],
                                              collapse = ", ")));
  if (sum(!gsg$goodSamples)>0)
    printFlush(paste("Removing samples:",
                     paste(rownames(datExpr0)[!gsg$goodSamples], collapse = ", ")));     Remove the
offending genes and samples from the data:
  datExpr0 = datExpr0[gsg$goodSamples, gsg$goodGenes]
}
gsg <- goodSamplesGenes(datExpr0,verbose = 3)
gsg$allOK


sampleTree = hclust(dist(datExpr0), method = "average")
par(cex = 0.6)
par(mar = c(0,4,2,0))
plot(sampleTree)
plot(sampleTree, main = "Sample clustering to detect outliers", sub="", xlab="", cex.lab = 1.5, cex.axis = 1.5,
cex.main = 2)
abline(h = 80, col = "red")
clust = cutreeStatic(sampleTree, cutHeight = 80, minSize = 10)
table(clust)
keepSamples = (clust==1)
datExpr0 = datExpr0[keepSamples, ]
dev.off()
sampleTree2 = hclust(dist(datExpr0), method = "average")
plot(sampleTree2)

nGenes = ncol(datExpr0)
nSamples = nrow(datExpr0)
save(datExpr0, nGenes, nSamples,file = "Step01-WGCNA_input.Rda")


enableWGCNAThreads()
powers = c(1:20)
sft = pickSoftThreshold(datExpr0, powerVector = powers, verbose = 5)

par(mfrow = c(1,2))
cex1 = 0.9
plot(sft$fitIndices[,1], -sign(sft$fitIndices[,3])*sft$fitIndices[,2],
     xlab="Soft Threshold (power)",ylab="Scale Free Topology Model Fit,signed R^2",type="n",
```

```r
        main = paste("Scale independence"));
text(sft$fitIndices[,1], -sign(sft$fitIndices[,3])*sft$fitIndices[,2],
        labels=powers,cex=cex1,col="red");
abline(h=0.90,col="red")
plot(sft$fitIndices[,1], sft$fitIndices[,5],
        xlab="Soft Threshold (power)",ylab="Mean Connectivity", type="n",
        main = paste("Mean connectivity"))
text(sft$fitIndices[,1], sft$fitIndices[,5], labels=powers, cex=cex1,col="red")

softPower =sft$powerEstimate #最佳 power 值
adjacency = adjacency(datExpr0, power = softPower)

TOM = TOMsimilarity(adjacency)
dissTOM = 1-TOM
save(TOM,file = "TOM.Rda")

geneTree = hclust(as.dist(dissTOM), method = "average");
plot(geneTree, xlab="", sub="", main = "Gene clustering on TOM-based dissimilarity",
        labels = FALSE, hang = 0.04)

minModuleSize = 30
dynamicMods = cutreeDynamic(dendro = geneTree, distM = dissTOM,
                            deepSplit = 2, pamRespectsDendro = FALSE,
                            minClusterSize = minModuleSize);
table(dynamicMods)

dynamicColors = labels2colors(dynamicMods)
table(dynamicColors)

plotDendroAndColors(geneTree, dynamicColors, "Dynamic Tree Cut",
                    dendroLabels = FALSE, hang = 0.03,
                    addGuide = TRUE, guideHang = 0.05,
                    main = "Gene dendrogram and module colors")

MEList = moduleEigengenes(datExpr0, colors = dynamicColors)
MEs = MEList$eigengenes
MEDiss = 1-cor(MEs);
METree = hclust(as.dist(MEDiss), method = "average")
plot(METree, main = "Clustering of module eigengenes",
        xlab = "", sub = "")

MEDissThres = 0.85
abline(h=MEDissThres, col = "red")
```

```
merge = mergeCloseModules(datExpr0, dynamicColors, cutHeight = MEDissThres, verbose = 3)
mergedColors = merge$colors
mergedMEs = merge$newMEs
plotDendroAndColors(geneTree, mergedColors,"Dynamic Tree Cut",
                    dendroLabels = FALSE, hang = 0.03,
                    addGuide = TRUE, guideHang = 0.05,
                    main = "Gene dendrogram and module colors")

moduleColors = mergedColors
table(moduleColors)
colorOrder = c("grey", standardColors(50))
moduleLabels = match(moduleColors, colorOrder)-1
MEs = mergedMEs
dev.off()
clinical <- read.table("clinical.txt",sep = "\t",row.names = 1,check.names = F,stringsAsFactors = F,header = T)
clinical <- clinical[rownames(datExpr0),]
identical(rownames(clinical),rownames(datExpr0))

head(clinical)
datTraits = as.data.frame(do.call(cbind,lapply(clinical, as.numeric)))
rownames(datTraits) = rownames(clinical)

sampleTree2 = hclust(dist(datExpr0), method = "average")

traitColors = numbers2colors(datTraits, signed = FALSE)

plotDendroAndColors(sampleTree2,
                    traitColors,
                    groupLabels = names(datTraits),
                    main = "Sample dendrogram and trait heatmap")
dev.off()

MEs=orderMEs(MEs)
moduleTraitCor=cor(MEs, datTraits, use="p")
write.table(file="Step04-modPhysiological.cor.xls",moduleTraitCor,sep="\t",quote=F)
moduleTraitPvalue=corPvalueStudent(moduleTraitCor, nSamples)
write.table(file="Step04-modPhysiological.p.xls",moduleTraitPvalue,sep="\t",quote=F)

textMatrix=paste(signif(moduleTraitCor,2),"\n(",signif(moduleTraitPvalue,1),")",sep="")
dim(textMatrix)=dim(moduleTraitCor)
labeledHeatmap(Matrix=moduleTraitCor,
               xLabels=colnames(datTraits),
               yLabels=names(MEs),
               ySymbols=names(MEs),
```

```
                              colorLabels=FALSE,
                              colors=blueWhiteRed(50),
                              textMatrix=textMatrix,
                              setStdMargins=FALSE,
                              cex.text=0.7,
                              cex.lab=0.7,
                              zlim=c(-1,1),
                              main=paste("Module-trait relationships"))
dev.off()

modNames = substring(names(MEs), 3)
geneModuleMembership = as.data.frame(cor(datExpr0, MEs, use = "p"))
a <- geneModuleMembership
a <- a %>% rownames_to_column()

MMPvalue = as.data.frame(corPvalueStudent(as.matrix(geneModuleMembership), nSamples))
names(geneModuleMembership) = paste("MM", modNames, sep="")
names(MMPvalue) = paste("p.MM", modNames, sep="")

traitNames=names(datTraits)
geneTraitSignificance = as.data.frame(cor(datExpr0, datTraits, use = "p"))
GSPvalue = as.data.frame(corPvalueStudent(as.matrix(geneTraitSignificance), nSamples))
names(geneTraitSignificance) = paste("GS.", traitNames, sep="")
names(GSPvalue) = paste("p.GS.", traitNames, sep="")

for (trait in traitNames){
  traitColumn=match(trait,traitNames)
  for (module in modNames){
    column = match(module, modNames)
    moduleGenes = moduleColors==module
    if (nrow(geneModuleMembership[moduleGenes,]) > 1){
      outPdf=paste(trait, "_", module,".pdf",sep="")
      pdf(file=outPdf,width=7,height=7)
      par(mfrow = c(1,1))
      verboseScatterplot(abs(geneModuleMembership[moduleGenes, column]),
                         abs(geneTraitSignificance[moduleGenes, traitColumn]),
                         xlab = paste("Module Membership in", module, "module"),
                         ylab = paste("Gene significance for ",trait),
                         main = paste("Module membership vs. gene significance\n"),
                         cex.main = 1.2, cex.lab = 1.2, cex.axis = 1.2, col = module)
      abline(v=0.8,h=0.5,col="red")
      dev.off()
    }
  }
```

```
}

for (mod in 1:nrow(table(moduleColors)))
{
   modules = names(table(moduleColors))[mod]
   probes = colnames(datExpr0)
   inModule = (moduleColors == modules)
   modGenes = probes[inModule]
   write.table(modGenes,
file =paste0(modules,".txt"),sep="\t",row.names=F,col.names=F,quote=F)
}
```

7.Machine Learning

```
#install.packages("glmnet")

#set.seed(1)
library(glmnet)
inputFile="normalize.txt"
geneFile="Hubgenes.txt"
setwd("lasso")

data=read.table(inputFile, header=T, sep="\t", check.names=F, row.names=1)

geneRT=read.table(geneFile, header=F, sep="\t", check.names=F)
data=data[as.vector(geneRT[,1]),]

data=t(data)
group=gsub("(.*)\\_(.*)", "\\2", row.names(data))
rt=as.data.frame(data)
rt$Type=ifelse(group=="Control", 0, 1)

x=as.matrix(rt[,1:(ncol(rt)-1)])
y=rt[,"Type"]
fit=glmnet(x, y, family = "binomial", alpha=1)
pdf(file="lasso.pdf",width=6,height=5.5)
plot(fit)
dev.off()

cvfit=cv.glmnet(x, y, family="binomial", alpha=1,type.measure='deviance',nfolds = 10)
pdf(file="cvfit.pdf",width=6,height=5.5)
plot(cvfit)
dev.off()
```

```
coef=coef(fit, s = cvfit$lambda.min)
index=which(coef != 0)
lassoGene=row.names(coef)[index]
lassoGene=lassoGene[-1]
write.table(lassoGene, file="lasso.gene.txt", sep="\t", quote=F, row.names=F, col.names=F)
outTab=rt[,c(lassoGene, "Type")]
outTab=cbind(id=row.names(outTab), outTab)
write.table(outTab, file="lasso.geneExp.txt", sep="\t", quote=F, row.names=F)
rm(list = ls())

library(randomForest)
set.seed(123456)
setwd("random forest")

inputFile="lasso.geneExp.txt"
data=read.table(inputFile, header=T, sep="\t", check.names=F, row.names=1)
#data=t(data)
data <- data[,1:4]
group=gsub("(.*)\\_", "\\7", row.names(data))

rf=randomForest(as.factor(group)~., data=data, ntree=2000)
pdf(file="RF.pdf", width=6, height=6)
plot(rf, main="Random forest", lwd=2)
dev.off()

optionTrees=which.min(rf$err.rate[,1])
optionTrees
rf2=randomForest(as.factor(group)~., data=data, ntree=optionTrees)
importance=importance(x=rf2)

pdf(file="GeneIm.pdf", width=6.2, height=5.8)
varImpPlot(rf2, main="")
dev.off()

rfGenes=importance[order(importance[,"MeanDecreaseGini"], decreasing = TRUE),]
rfGenes=names(rfGenes[rfGenes>6])
#rfGenes=names(rfGenes[1:30])
write.table(rfGenes, file="Genes.txt", sep="\t", quote=F, col.names=F, row.names=F)
sigExp=t(data[,rfGenes])
sigExpOut=rbind(ID=colnames(sigExp),sigExp)
write.table(sigExpOut, file="imGeneExp.txt", sep="\t", quote=F, col.names=F)
```

8.Immune Infiltration Analysis

```
library(e1071)

setwd("Immune")
inputFile="normalize.txt"
source("pathwayMR20.CIBERSORT.R")

outTab=CIBERSORT("ref.txt", inputFile, perm=1000, QN=T)

outTab=outTab[outTab[,"P-value"]<0.05,]
outTab=as.matrix(outTab[,1:(ncol(outTab)-3)])
outTab=rbind(id=colnames(outTab),outTab)
write.table(outTab, file="CIBERSORT-Results.txt", sep="\t", quote=F, col.names=F)

rm(list = ls())
library(reshape2)
library(ggpubr)
inputFile="CIBERSORT-Results.txt"
setwd("34.DCM-CIBERSORT")

rt=read.table(inputFile, header=T, sep="\t", check.names=F, row.names=1)

con=grepl("_Control", rownames(rt), ignore.case=T)
treat=grepl("_NAFLD", rownames(rt), ignore.case=T)
conData=rt[con,]
treatData=rt[treat,]
conNum=nrow(conData)
treatNum=nrow(treatData)
data=t(rbind(conData,treatData))

pdf(file="barplot.pdf", width=15, height=8)
col=rainbow(nrow(data), s=0.7, v=0.7)
par(las=1,mar=c(8,5,4,16),mgp=c(3,0.1,0),cex.axis=1.5)
a1=barplot(data,col=col,xaxt="n",yaxt="n",ylab="Relative Percent",cex.lab=1.8)
a2=axis(2,tick=F,labels=F)
axis(2,a2,paste0(a2*100,"%"))
par(srt=0,xpd=T)
rect(xleft = a1[1]-0.5, ybottom = -0.01, xright = a1[conNum]+0.5, ytop = -0.06,col="#008B45FF")
text(a1[conNum]/2,-0.035,"Control",cex=1.8)
rect(xleft = a1[conNum]+0.5, ybottom = -0.01, xright =a1[length(a1)]+0.5, ytop = -0.06,col="#EE0000FF")
text((a1[length(a1)]+a1[conNum])/2,-0.035,"NAFLD",cex=1.8)
ytick2 = cumsum(data[,ncol(data)])
ytick1 = c(0,ytick2[-length(ytick2)])
legend(par('usr')[2]*0.98,par('usr')[4],legend=rownames(data),col=col,pch=15,bty="n",cex=1.2)
dev.off()
```

```
Type=gsub("(.*)\\_(.*)", "\\2", rownames(rt))
data=cbind(as.data.frame(t(data)), Type)
data=melt(data, id.vars=c("Type"))
colnames(data)=c("Type", "Immune", "Expression")
group=levels(factor(data$Type))
bioCol=c("#008B45FF","#EE0000FF","#0066FF","#FF0000","#6E568C","#7CC767","#223D6C","#D20A13","#
FFD121","#088247","#11AA4D")
bioCol=bioCol[1:length(group)]
boxplot=ggboxplot(data, x="Immune", y="Expression", fill="Type",
                  xlab="",
                  ylab="Fraction",
                  legend.title="Type",
                  notch=T,
                  #add="point",
                  width=0.8,
                  palette=bioCol)+
  rotate_x_text(50)+
  stat_compare_means(aes(group=Type),symnum.args=list(cutpoints=c(0, 0.001, 0.01, 0.05, 1), symbols=c("***",
"**", "*", "")), label="p.signif")
pdf(file="immune.diff.pdf", width=8, height=6)
print(boxplot)
dev.off()
```

9.Single-cell Analysis

```
library(Seurat)
library(dplyr)

sc_data <- Read10X(data.dir = "path")
seurat_obj <- CreateSeuratObject(counts = sc_data, min.cells = 3, min.features = 200)

seurat_obj[["percent.mt"]] <- PercentageFeatureSet(seurat_obj, pattern = "^MT-")
seurat_obj <- subset(seurat_obj, subset = nFeature_RNA > 200 & nFeature_RNA < 6000 & percent.mt < 10)

seurat_obj <- NormalizeData(seurat_obj)
seurat_obj <- FindVariableFeatures(seurat_obj, selection.method = "vst", nfeatures = 2000)
seurat_obj <- ScaleData(seurat_obj, vars.to.regress = c("nCount_RNA","percent.mt"))
seurat_obj <- RunPCA(seurat_obj, npcs = 30)
seurat_obj <- FindNeighbors(seurat_obj, dims = 1:10)
seurat_obj <- FindClusters(seurat_obj, resolution = 0.5)
seurat_obj <- RunTSNE(seurat_obj, dims = 1:10)
seurat_obj <- RunUMAP(seurat_obj, dims = 1:10)

genes_of_interest <- c("CEBPD","THBS1","SOCS2","IFIT2")
```

```
DotPlot(seurat_obj, features = genes_of_interest) + RotatedAxis()
```
